# Efficient Methods for Parameter Estimation of Ordinary and Partial Differential Equation Models of Viral Hepatitis Kinetics

**DOI:** 10.3390/math8091483

**Published:** 2020-09-02

**Authors:** Alexander Churkin, Stephanie Lewkiewicz, Vladimir Reinharz, Harel Dahari, Danny Barash

**Affiliations:** 1 Department of Software Engineering, Sami Shamoon College of Engineering, Beer-Sheva 8410501, Israel; 2 Department of Mathematics, University of California at Los Angeles, Los Angeles, CA 90095, USA;; 3 Department of Computer Science, Université du Québec à Montréal, Montreal, QC H3C 3P8, Canada;; 4 Program for Experimental and Theoretical Modeling, Division of Hepatology, Department of Medicine, Stritch School of Medicine, Loyola University Medical Center, Maywoood, IL 60153, USA;; 5 Department of Computer Science, Ben-Gurion University, Beer-Sheva 8410501, Israel

**Keywords:** parameter estimation, constrained optimization, derivative free optimization, multiscale models, differential equations, viral hepatitis

## Abstract

Parameter estimation in mathematical models that are based on differential equations is known to be of fundamental importance. For sophisticated models such as age-structured models that simulate biological agents, parameter estimation that addresses all cases of data points available presents a formidable challenge and efficiency considerations need to be employed in order for the method to become practical. In the case of age-structured models of viral hepatitis dynamics under antiviral treatment that deal with partial differential equations, a fully numerical parameter estimation method was developed that does not require an analytical approximation of the solution to the multiscale model equations, avoiding the necessity to derive the long-term approximation for each model. However, the method is considerably slow because of precision problems in estimating derivatives with respect to the parameters near their boundary values, making it almost impractical for general use. In order to overcome this limitation, two steps have been taken that significantly reduce the running time by orders of magnitude and thereby lead to a practical method. First, constrained optimization is used, letting the user add constraints relating to the boundary values of each parameter before the method is executed. Second, optimization is performed by derivative-free methods, eliminating the need to evaluate expensive numerical derivative approximations. The newly efficient methods that were developed as a result of the above approach are described for hepatitis C virus kinetic models during antiviral therapy. Illustrations are provided using a user-friendly simulator that incorporates the efficient methods for both the ordinary and partial differential equation models.

## 1. Introduction

Chronic viral hepatitis (hepatitis C, hepatitis B, and hepatitis D) is a major public health concern. Approximately 500 million individuals worldwide are living with chronic viral hepatitis; above a million of those who are infected die each year, primarily from cirrhosis or liver cancer resulting from their hepatitis infection [1–3]. Deaths related to chronic hepatitis are as many as those due to human immunodeficiency virus (HIV) infection, tuberculosis, or malaria [4], and are projected to exceed the combined mortality associated with HIV infection, tuberculosis, and malaria by 2040 [5]. Only a small subset of patients are cured with currently available drugs for hepatitis B and hepatitis D. As such, a deeper understanding of hepatitis B and D infection dynamics is needed to enable the development of more curative therapeutics. Despite the significant advances in hepatitis C therapy, it is widely acknowledged that cost remains a major barrier for achieving global elimination. Thus, there still exists a need for affordable therapy with similar high efficacy and with much shorter treatment durations and vaccine development.

Mathematical models have been developed to provide insights into viral hepatitis and host dynamics during infection and the pathogenesis of infection [6–11]. The standard biphasic model for viral infection is a set of three ordinary differential equations (ODEs) with three variables. This ODE model has been used to study hepatitis C virus (HCV), hepatitis B virus (HBV), and hepatitis D virus (HDV) kinetics during antiviral treatment. It contributed to the assessment of antivirals efficacy and to our understanding of their mechanism of action [9,12–18]. The ODE model can be further simplified (termed biphasic mode) by assuming that target cells remain constant during antiviral treatment. The biphasic model has been extensively used for modeling HCV [19–27], HBV [28–31], or HDV [32–35] kinetics during antiviral treatment. Notably, we recently showed in a proof-of-concept pilot study that using the biphasic model in real time (i.e., on treatment) can shorten HCV treatment duration (and cost) with direct-acting antivirals without compromising efficacy or patient safety [36], which confirmed our retrospective biphasic modeling reports in more than 250 patients [37–41].

Modeling efforts using ODEs for understanding the intracellular viral hepatitis genome dynamics have been done in [7,42–46]. Recently, partial differential equation (termed PDE, age-structured or multiscale) models for HCV infection and treatment were developed [47–50]. These PDE models are an extension to the classical biphasic models in which the infected cell is a “black box”, producing virions but without any consideration of the intracellular viral RNA replication and degradation within the infected cell [42,43,51]. The multiscale models consider the intracellular viral RNA in an additional equation for the variable (R), with the introduction of age-dependency in addition to time-dependency, making it a PDE model. They are considerably more difficult to solve and to perform parameter estimation on compared to the biphasic model. Unlike the construction of numerical schemes in other applications, for example in the nonlinear diffusion of digital images [52–54] where accuracy can be limited, herein it is advisable to construct a stable and efficient scheme that belongs to the Runge–Kutta family with a higher accuracy than in nonlinear diffusion. Our numerical solution strategy was outlined in [55–57] and herein we continue [57] by providing an efficient parameter estimation method that follows this strategy.

Parameter estimation (or calibration) of multiscale HCV models with HCV kinetic data measured in treated patients is challenging. To overcome this, several strategies have been employed. The first strategy, employed in [48], utilizes an analytical solution named long-term approximation for solving the model equations along with calling the Levenberg–Marquardt [58,59] as a canned method for performing the fitting. The second strategy, employed in [60], transforms the multiscale model to a system of ODEs and, as such, simple parameter estimation methods can be used in the same manner as the biphasic model. The third strategy, employed in [50] that also deals with spatial models of intracellular virus replication, is based on the method of lines and utilizes canned methods for both the numerical solution of the resulting equations (Matlab’s *ode45*) and for performing the fitting (Matlab’s *fmincon*). While these strategies are adequate for specific cases, they rely on canned methods and are problematic when it comes to the user’s capability to access and control them. For these reasons, we have developed our own open source code released free of charge for the benefit of the community that allows the user to make modifications to the model and provides prospects for future development, while ensuring that it is practical in running time and enabling the user to insert constraints for the parameters that need to be estimated. In contrast to these approaches, our strategy does not rely on any canned method but fully implements our own optimization routine, thus making it suitable to other multiscale model equations by modifications inside the routine and an early preparation of the multiscale model equations by taking derivatives with respect to the parameters before the optimization procedure.

The general ideas that have led to [57], including the parameter estimation procedure described in this reference, have been laid out in order to remain self-contained. The motivation of the present work is to develop a tool that can provide similar calibration values in significantly less time. More specifically, the main contribution herein is as follows. Because of precision problems in [57] encountered with Levenberg–Marquardt that caused the parameter estimation procedure to become highly non-efficient, we developed an efficient constrained optimization procedure that is based on damped Gauss–Newton instead such that we avoid problematic use of derivatives, while alternatively offering the possibility to apply Powell’s Constrained optimization by linear approximation (COBYLA) [61] for the optimization procedure. In the following sections, we describe the model and the optimization procedure that is used in our HCVMultiscaleFit simluator. Illustrations of simultations using HCVMultiscaleFit are provided and the efficiency and practicality relative to the initial version put forth in [57] are discussed.

## 2. Methods

### 2.1. Development of Mathematical Models

#### 2.1.1. The Standard Biphasic Model

The three variables this model keeps track of are the target cells *T*, in Equation (1), the infected cells *I* in Equation (2), and the free virus *V* in Equation (3). The target cells *T* are produced at constant rate *s*, die at per capita rate *d*, and are infected by virus *V* at constant rate *β*. The infected cells *I* increase with the new infections at rate *βV*(*t*)*T*(*t*) and die at constant rate *δ*. The virus *V* is produced at rate *p* by each infected cell and is cleared at constant rate *c*. The *ϵ* term denotes the effectiveness of the anti-viral treatment that decreases the production from *p* to (1 − *ϵ*)*p*. Formally, the ensemble of ODEs for this model is:

(1) $$\frac{\text{d}T(t)}{\text{d}t}=s-\beta V(t)T(t)-dT(t)$$

(2) $$\frac{\text{d}I(t)}{\text{d}t}=\beta V(t)T(t)-\delta I(t)$$

(3) $$\frac{\text{d}V(t)}{\text{d}t}=(1-\epsilon )pI(t)-cV(t).$$

From the mathematical perspective, the standard biphasic model is relatively much simpler than the multiscale model. Although it is nonlinear, it can be solved analytically when assuming that *T* is constant (target cells remain constant during antiviral treatment).

#### 2.1.2. The Multiscale HCV Model

A multiscale PDE model for HCV infection and treatment dynamics was introduced in [47–49]. Intracellular HCV RNA plays a biologically significant role during the HCV replication and multiscale models are considering it by additional equations for the RNA that are age-dependent, with the most complete model to date that was recently put forth in [50].

The multiscale model [47–49] can be formulated as follows:

(4) $$\frac{\text{d}T(t)}{\text{d}t}=s-dT(t)-\beta V(t)T(t)$$

(5) $$\frac{\partial I(a,t)}{\partial t}+\frac{\partial I(a,t)}{\partial a}=-\delta I(a,t)$$

(6) $$\frac{\text{d}V(t)}{\text{d}t}=\left(1-\varepsilon_{s}\right)\int_{0}^{\infty }\rho R(a,t)I(a,t)\text{d}a-cV(t)$$

(7) $$\frac{\partial R(a,t)}{\partial t}+\frac{\partial R(a,t)}{\partial a}=\left(1-\varepsilon_{\alpha }\right)\alpha {\text{e}}^{-\gamma t}-\left(\left(1-\varepsilon_{s}\right)\rho +\kappa \mu \right)R(a,t),$$

with the initial and boundary conditions $T(0)=\bar{T},V(0)=\bar{\nabla },I(0,t)=\beta V(t)T(t),I(a,0)=\bar{I}(a),R(0,t)=1$, and $R(a,0)=\bar{R}(a)$. The initial condition *R*(0, *t*) = 1 reflects the assumption that a cell is infected by a single virion and therefore there is only one vRNA in an infected cell at age zero.

The four variables this model keeps track of are the target cells *T* in Equation (4), the infected cells *I* in Equation (5), the free virus *V* in Equation (6), and the intracellular viral RNA *R* in an infected cell in Equation (7).

The target cells *T* are produced at constant rate *s*, and decrease by the number of cells infected by virus in blood *V* at constant rate *β* and their death rate *d*. The infected cells *I* die at constant rate *δ*. The quantity of intracellular viral RNA *R* depends on its production *α* and its degradation *μ* and expulsion from the cell *ρ*. The quantity of free virus *V* depends on the number of assembled and released virions and their clearance rate *c*. The parameter *γ* represents the decay of replication template under therapy. The decrease in viral RNA synthesis is represented by *ε*_*α*_, the reduction in secretion by *ε*_*s*_, and the increase in viral degradation by *κ* ≥ 1.

The parameters that were used in the multiscale model described in [48] are depicted in Table 1. The model forms an example of our parameter estimation calibration method for PDE models developed herein that can easily be extended to include additional parameters.

An important consideration in this model is that the treatment starts after the infection has reached its steady state. The steady states of the different variables are $\bar{R}(a,t)$, $\bar{I}(a,t)$, $\bar{V}$, and $\bar{T}$. The term *N* represents the total number of virions produced by infected cells.

These values have been previously derived in [48] and can be expressed as follows:

(8) $$\bar{T}=c/\beta N$$

(9) $$\bar{V}=(\beta Ns-dc)/(\beta c)$$

(10) $$\bar{I}(a)=\beta \bar{V}\bar{T}{\text{e}}^{-\delta a}$$

(11) $$\bar{R}(a)=\frac{\alpha }{\rho +\mu }+\left(1-\frac{\alpha }{\rho +\mu }\right){\text{e}}^{-(\rho +\mu )(a)}$$

(12) $$N=\frac{\rho (\alpha +\delta )}{\delta (\rho +\mu +\delta )}$$

It has been shown that the equations for *I*(*a*, *t*) and *R*(*a*.*t*) can be solved by the method of characteristics to yield:

(13) $$I(a,t)=\{\begin{array}{ll} \beta V(t-a)T(t-a){\text{e}}^{-\delta a} & a<t\\ \bar{I}(a-t){\text{e}}^{-\delta t}=\beta \bar{V}\bar{T}{\text{e}}^{-\delta (t-a)}=(\beta Ns-dc)/(\beta N){\text{e}}^{-\delta a} & a>t \end{array}$$

and

(14) $$R(a,t)=\left\{\begin{array}{ll} \frac{\left(1-\varepsilon_{\alpha }\right)\alpha {\text{e}}^{-\gamma t}}{\left(1-\varepsilon_{s}\right)\rho +\kappa \mu -\gamma }+\left(1-\frac{\left(1-\varepsilon_{\alpha }\right)\alpha {\text{e}}^{-\gamma (t-a)}}{\left(1-\varepsilon_{s}\right)\rho +\kappa \mu -\gamma }\right){\text{e}}^{-\left(\left(1-\varepsilon_{s}\right)\rho +\kappa \mu \right)a} & a<t\\ \frac{\left(1-\varepsilon_{\alpha }\right)\alpha {\text{e}}^{-\gamma t}}{\left(1-\varepsilon_{s}\right)\rho +\kappa \mu -\gamma }+\left(\frac{\alpha }{\rho +\mu }+\left(1-\frac{\alpha }{\rho +\mu }\right){\text{e}}^{-(\rho +\mu )(a-t)}-\frac{\left(1-\varepsilon_{\alpha }\right)\alpha }{\left(1-\varepsilon_{s}\right)\rho +\kappa \mu -\gamma }\right){\text{e}}^{-\left(\left(1-\varepsilon_{s}\right)\rho +\kappa \mu \right)t} & a>t \end{array}\right.$$

whereas the equations for *V*(*a*, *t*) and *T*(*a*, *t*) cannot be solved analytically without any approximations. The equations for *V*(*a*, *t*) and *T*(*a*, *t*) when using the short-term and long-term approximations can be found in [48].

### 2.2. Data Description

Calibration of the model was performed with data from treated patients by [48]. The data points to fit the model and on which the error is computed are only *V*. We assume that we start at a steady state and begin by computing the steady state given the initial parameters by using Equation (8). While the raw data are not available, we used the freely accessible tool of [62] to retrieve it from the figures directly. A visual example for one patient is available at [57].

In our method, we mostly use the default parameters from [48] that are shown in Table 2. The main difference concerns parameter *s*. The pre-treatment steady state viral load $\bar{V}$ in each patient is different. Since $\bar{V}$ is a necessary value in computing the long-term approximation, it was approximated as the pre-treatment viral load observed per patient. In the full model that we are implementing, we do not directly use $\bar{V}$. Instead, we have from Equation (9) that $\bar{V}$ is a function of many parameters, in particular *s* which is not present in the long term approximation that was outlined in [48]. Inspired by the method of [48], we chose to also fix $\bar{V}$. The counterpart in our method is that *s* changes per patient being, by Equation (9), equal to $\left(\bar{V}\beta c+dc)/(\beta N\right)$, where *N* is from Equation (12).

More details about preparing the system with data from patients and the model parameters are available in [57]. Herein, the methods are different from [57] and are significantly more efficient, but the model parameters and the system preparation are exactly the same.

### 2.3. Solving the Model Equations

In [48], the multiscale model equations were solved by analytical approximations but, as discussed in [56], those analytical approximations have limitations that should be alleviated. The long-term approximation is an underestimate of the PDE model since some infection events are being ignored. Moreover, for each multiscale model, the long-term approximation needs to be derived analytically, which is not a trivial task. Thus, numerical solutions provide an attractive alternative and could be easier to adjust when introducing changes to the model. A more general and comprehensive approach to parameter fitting without relying on analytical approximations would be useful. In addition, although it was shown recently that it is possible to transform the PDE multiscale model to a system of ODEs [60], this transformation problematically introduces some of the boundary conditions, e.g., *ζ*, as new parameters inside the model equations. A numerical approach to parameter fitting of multiscale models was recently put forth and described in [50], by the use of the method of lines and canned methods that are available in Matlab. Our new numerical approach that originated in [56] and described in [57] in detail does not rely on canned methods, with considerable benefits.

For the numerical solution of the multiscale model equations, properties such as approximation, stability, and convergence were discussed in [56] and numerical robustness was discussed in [55,56]. Future work should expand towards the advanced treatment of properties as covered in [63,64]. Concerning the numerical solution itself, we showed in [56] that the full implementation of the Rosenbrock method is preferable over the use of a canned solver in terms of efficiency and stability. Therefore, the Rosenbrock method has been implemented for the purpose of our parameter fitting method as well. In order to apply the Rosenbrock method, it is simplest to represent the system to be solved as a vector *f* of two functions:

(15) $$y^{\prime }=f(t,y)={\left[\frac{\text{d}T}{\text{d}t},\frac{\text{d}V}{\text{d}t}\right]}^{⊤}=[s-dT-\beta VT,\;{\left(1-\varepsilon_{s}\right)\int_{0}^{\infty }\rho R(a,t)I(a,t)\text{d}a-cV]}^{⊤},$$

where *y* is a vector with the values of [*T*, *V*]^⊤^ and the transpose symbol can be omitted from now on for brevity. This representation has originated in [56] for convenience with formulating the numerical schemes described in that reference. This function depends on three variables, *t*, *V* and *T*. While *V* and *T* are the values at the time point we are evaluating, inside the equation of *I*, the function *V*(*t* − *a*) and *T*(*t* − *a*) do depend on *t* directly. In our implementation, when computing the integral, we need to divide into two cases. If *a* > *t*, we analytically determine the values of *R*(*a*, *t*) and *I*(*a*, *t*) for small time steps *a*. When *a* < *t*, the system was previously solved at times *τ*_0_, …, *τ*_*n*_. Therefore, we evaluate the integrals at times *a*_0_ = *t* − *τ*_0_, …, *a*_*n*_ = *t* − *τ*_*n*_, ensuring that the required values of *V*(*t* − *a*) and *T*(*t* − *a*) are already known, following the scheme presented in [56].

The Rosenbrock method additionally requires the Jacobian matrix, denoted by *f*′. As was shown in [57], the Jacobian can be controlled and, with some proper computational simplifications to avoid singularities that were shown to yield correct results in [57], we can implement the Rosenbrock method convincingly for both solution and parameter estimation of the multiscale models.

### 2.4. Parameter Estimation

#### 2.4.1. Preliminaries

As outlined in [48], the HCV multiscale model has 12 parameters (Table 1) and the nonlinear differential equations that comprise it are stiff [56]. In addition, the integral term in the equation complicates matters, as described in [56,57]. Parameter fitting is known to be a difficult problem in general and for multiscale models, in particular, one needs to approach it carefully with the use of robust techniques for the optimization, but, at the same time, these techniques can be made highly efficient for practical computations. The novelty in this work is described next.

For efficiency reasons, we revert from the Levenberg–Marquardt method for optimization that was used in albeit different ways in both [48,57] and implement significant improvements. Already in [57], we have noticed that more difficult fitting cases take several hours to perform, and this situation needs to be remedied for a practical use of our simulator. The reason for the lengthy running times was non-trivial and only after a considerable period of time, having tried the simplest numerical method for the solution of the equations (the Euler method instead of the Rosenbrock method) and not noticing a significant time reduction in the parameter estimation calculation, we began to understand that the problem lies in the optimization method being used. We then examined interior point methods for performing constrained optimization instead of the Levenberg–Marquardt method we used in [57] and found out that the Hessian calculations in these interior point methods are problematic, causing precision problems near the parameter boundaries that are the source of running time accumulations. There was definitely a need to avoid the use of derivatives and therefore two alternative approaches were taken. The first was to try a constrained damped Gauss–Newton strategy, which can also be looked at as a simple version of Levenberg–Marquardt without gradient descent, or alternatively Levenberg–Marquardt is a pseudo second-order method with added derivatives to approximate the Hessian and thereby adds complications that should better be avoided. While in general Levenberg–Marquardt is considered more robust than Gauss–Newton, for our constrained application, the simplicity of the damped Gauss–Newton in terms of derivative calculations relative to Levenberg–Marquardt, in which also the Lagrange parameter needs to be calculated at each step, makes the damped Gauss–Newton significantly preferable. The second approach taken was that, while developing our own damped Gauss–Newton method for the constrained application, we also examined a completely derivative-free approach based on COBYLA (Constrained Optimization by Linear Approximation). These two approaches turned out to be complementary to each other as by default the quicker and sometimes somewhat more accurate damped Gauss–Newton can be tried first, but, when it fails, COBYLA can provide a good alternative or it can even be used from the start and all along a research study as the difference in the calculated error that has been minimized is quite small. This contribution allows for reaching an overall procedure for parameter estimation that is practical and by orders of magnitude less demanding in computing time relative to [57], which provides a technical breakthrough from the computational standpoint.

**Figure 1.**
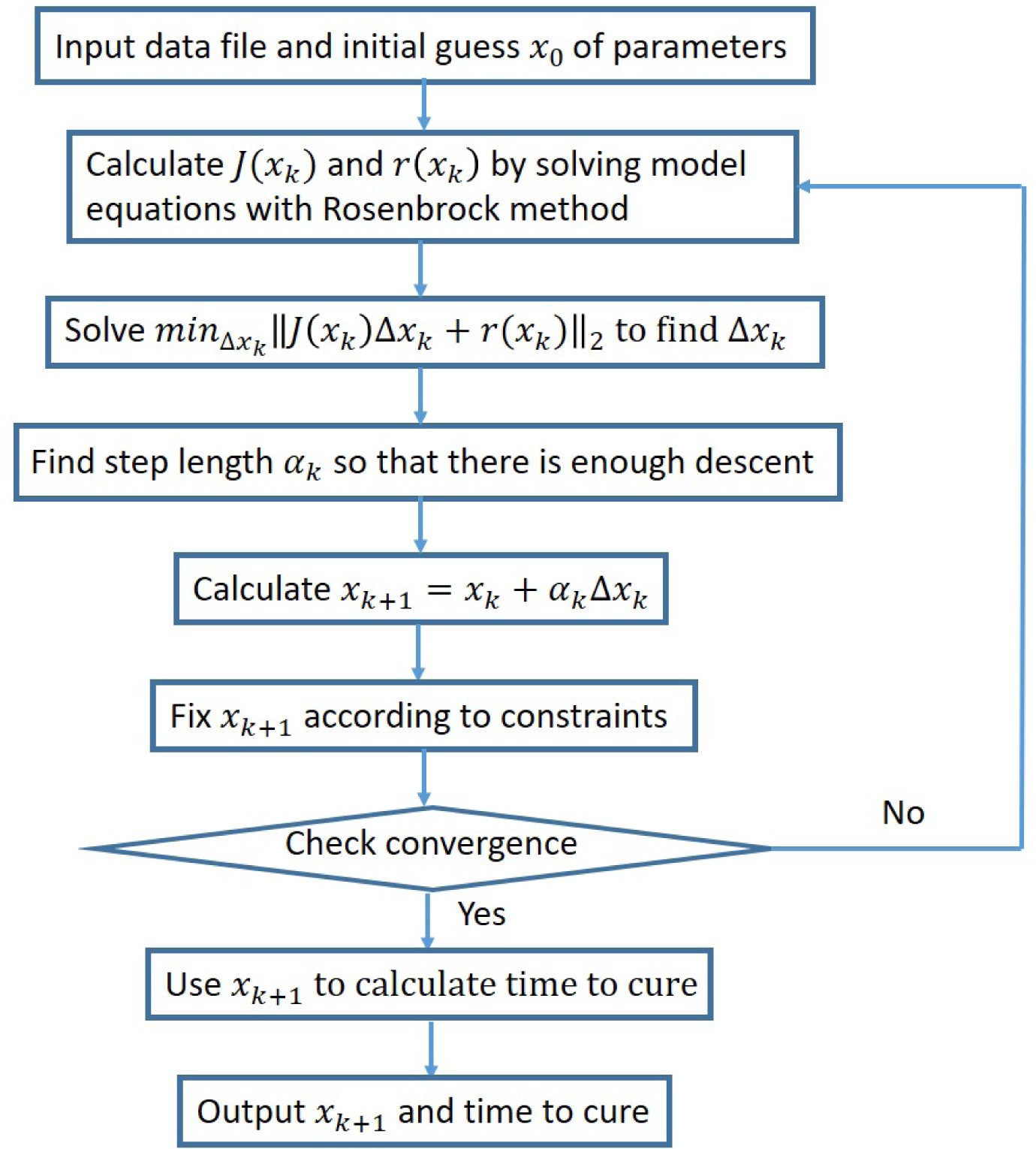
A flowchart of our constrained damped Gauss–Newton method.

Thus, two newly developed methods have been introduced to perform constrained optimization for this application in an efficient manner: LSF (Least Squares Fitter using Gauss–Newton) with a flowchart shown in Figure 1 and Powell’s COBYLA (Constrained Optimization by Linear Approximation) with a pseudocode shown in Algorithm 1. The latter is a derivative-free optimization method that solves the constrained optimization by linear programming. The former is a constrained optimization that performs linearization in the manner described herein.

In both approaches, the objective function to be minimized is described as follows. The objective consists of adjusting the parameters of a model function to best fit a data set. A simple data set consists of *n* points (data pairs) (*x*_*i*_, *y*_*i*_), *i* = 1, …, *n*, where *x*_*i*_ is an independent variable and *y*_*i*_ is a dependent variable whose value is found by observation. The model function has the form *f*(*x*, *p*), where *m* adjustable parameters are held in the vector *p*. The goal is to find the parameter values for the model that best fits the data. The fit of a model to a data point is measured by its residual, defined as the difference between the actual value of the dependent variable and the value predicted by the model:

(16) $$r_{i}=y_{i}-f\left(x_{i},p\right)$$

The least-squares method obtains the optimal parameter values by minimizing the sum of squared residuals:

(17) $$S=\overset{n}{\underset{i=1}{\sum }}{\left(r_{i}\right)}^{2}=\overset{n}{\underset{i=1}{\sum }}{\left(y_{i}-f\left(x_{i},p\right)\right)}^{2}$$

#### 2.4.2. Optimization by a Constrained Version of Nonlinear Least Squares (Gauss–Newton Method)

If we assume that *f*(*x*) is twice continuously differentiable, then we can utilize Newton’s method to solve the system of nonlinear equations:

(18) $$\nabla f(x)=J{\left(x\right)}^{T}r(x)=0,$$

which provides local stationary points for *f*(*x*), where *r*(*x*) is the vector of residuals associated with data points as functions of parameter vector *x* and *J* is the Jacobian. Written in terms of derivatives of *r*(*x*) and starting from an initial guess *x*_0_, this version of the Newton iteration scheme takes the form:

(19) $$x_{k+1}=x_{k}-{\left[J{\left(x_{k}\right)}^{T}J\left(x_{k}\right)+S\left(x_{k}\right)\right]}^{-1}J{\left(x_{k}\right)}^{T}r\left(x_{k}\right),\;k=0,1,2,\ldots$$

where *S*(*x*_*k*_) denotes the matrix:

(20) $$S\left(x_{k}\right)=\sum_{i=1}^{m}r_{i}\left(x_{k}\right)\nabla^{2}r_{i}\left(x_{k}\right).$$

In order to obtain the correction Δ*x*_*k*_ = *x*_*k*+1_ − *x*_*k*_, a linear system is solved by a direct or iterative method:

(21) $$\left[J{\left(x_{k}\right)}^{T}J\left(x_{k}\right)+S\left(x_{k}\right)\right]\Delta x_{k}=-J{\left(x_{k}\right)}^{T}r\left(x_{k}\right).$$

For our application, we use the Gauss–Newton method, which neglects the second term *S*(*x*_*k*_) of the Hessian, and the computation of the step Δ*x*_*k*_ involves the solution of the linear system:

(22) $$\left[J{\left(x_{k}\right)}^{T}J\left(x_{k}\right)\right]\Delta x_{k}=-J{\left(x_{k}\right)}^{T}r\left(x_{k}\right),$$

and *x*_*k*+_1 = *x*_*k*_ + Δ*x*_*k*_.

In our application, we use the following steps that comprise a damped Gauss–Newton strategy:

- Start with an initial guess *x*_0_ and iterate for *k* = 0, 1, 2, …
- Solve ${\text{min}}_{\Delta x_{k}}{\left\|J\left(x_{k}\right)\Delta x_{k}+r\left(x_{k}\right)\right\|}_{2}$. to compute the correction Δ*x*_*k*_.
- Choose a step length *α*_*k*_ so that there is enough descent.
- Calculate the new iterate *x*_*k*+1_ = *x*_*k*_ + *α*_*k*_Δ*x*_*k*_.
- Check for convergence.

We choose *α*_*k*_ to be 1.0 at the beginning of the algorithm and decrease it by dividing by two each time the error increases relative to the previous iteration. More sophisticated damping strategies such as the Armijo–Goldstein step-length principle are not suitable in our application because of constraints violation that is described next. We also extend the Damped Gauss–Newton method to be constrained in the following way: in the case that one of the parameters, during the convergence process, exceeds its bounds (constraints provided in the GUI by the user), the algorithm assigns to this parameter its corresponding bound value instead. For this reason, we cannot apply the Armijo–Goldstein condition and need to revert to a simple damping strategy that is suitable with our constrained modification of a damped Gauss–Newton method. A flowchart of our method is shown in Figure 1.

#### 2.4.3. Optimization by Derivative-Free Methods (COBYLA Method)

Should the Gauss–Newton method fail to carry out the optimization of Equation (18), a helpful alternative is the COBYLA algorithm, a derivative-free simplex method originally developed by Powell [65]. The parameters in the algorithm have mathematical meanings that are outside the scope of the model employed, as will be shown herein, and a pseudocode of the algorithm is available in Algorithm 1. In general, a simplex method seeks to minimize an objective function using *simplices*, where *simplex* refers to the convex hull of a set of *n* + 1 points in *n*-dimensional space. Such an algorithm begins by evaluating the objective function at the vertices of an initial simplex, and then strategically adjusting the simplex so that the objective function attains generally smaller values at the vertices of the new simplex than it did at those of the previous simplex. At each iteration, a vertex of the simplex may be altered, or the simplex itself rescaled, so as to guide the simplex into a region at which the objective function is minimized. When sufficient accuracy is attained, the vertex of the final simplex at which the objective function is smallest is returned as the function’s minimizer.

A major benefit of both the Gauss–Newton and COBYLA algorithms is in reducing and even abolishing the use of derivatives of the objective function. In our model, the Hessian matrix associated with our objective function imposes a heavy computational burden on the optimization problem, and methods that do not require it are preferable. Numerical results indicate that COBYLA is generally very effective when the Gauss–Newton method fails; the latter, however, is quicker and more accurate than COBYLA. By default, we use the Gauss–Newton method, and, when it fails, the user is prompted to initiate COBYLA. The details of the COBYLA algorithm are described in Appendix A, beginning with a description of the Nelder and Mead simplex method from which it is derived.

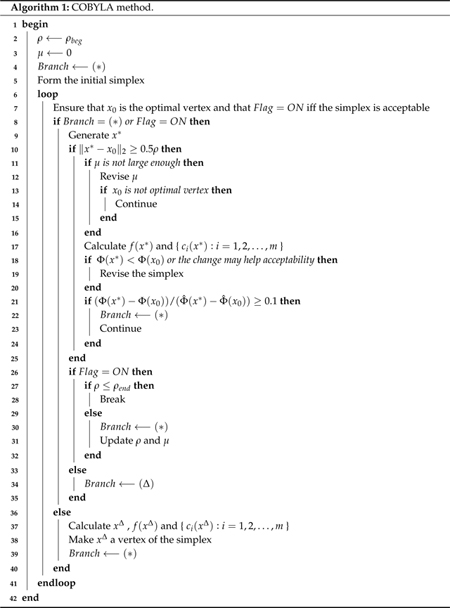



### 2.5. Method Scope and Other Approaches

The strategy that was introduced in [57] and also implemented herein prepares the multiscale model equations for parameter fitting by working on them directly as an initial step. This strategy is beneficial in postponing approximations to later steps and ensuring full control of the user during the whole fitting procedure. It should be noted that, for each parameter introduced in future multiscale models, the derivative with respect to the new parameter needs to be taken and more equations need to be derived, as illustrated in this section. However, this technical procedure is significantly less complicated than deriving analytical approximations to a modified model with a change in the parameters. In our package, the code is written in Java and at present the method is hard coded for the model; therefore, some technical expertise is needed if a new model is given and the method needs to be hard coded in Java for the new model. In future work we plan to separate the model from the method and make it generic, which needs to be done only once and then it can easily handle various modifications to the model and become modeler friendly. Until that time, we do rely on some amount of expert knowledge, but, overall, it should still be easier than deriving analytical approximations to a modified model.

The importance of parameter estimation to the model was already noted in previous studies. It was addressed in [48] and attempts to come up with improved strategies were tried thereafter in [60] and in [50]. Here, we briefly relate to each of these approaches in order to remain self-contained. More information can be found in [57].

#### 2.5.1. Parameters Change When Transforming a PDE Multiscale Model to a System of ODEs

An approach taken in [60] showed how a PDE multiscale model of hepatitis C virus can be transformed to a system of ODEs. In principle, parameter estimation should then become easier, avoiding the complications in dealing with the PDE multiscale model. However, there are side effects introduced in such a transformation, as can be noticed in Equation (9) of [60] where the boundary condition *R*(*t*, 0) = *ζ* gets inside the differential equations. Consequently, as admitted in the discussion of that reference, all parameters in Equations (7)–(10) must be estimated including *ζ*. The inclusion of boundary conditions as new parameters inside the model equations is a drawback compared to parameter estimation performed on the original multiscale model equations before the transformation. Another drawback from the perspective of parameters change is the fact that the simplest PDE multiscale model appearing in [47] was used in the transformation to ODEs, but important additions such as the inclusion of parameter *γ* as in [48] are not taken into account. It is not obvious how to include the parameter *γ* and other developments to the multiscale model inside the system of ODEs. Finally, any information regarding the age of the cell since infection is lost. Thus, if one would wish, for example, to vary the parameter *α* from infection to a certain time; this is not possible. In summary, while the transformation works for the simplest multiscale model, it is limited in considering developments to the multiscale model and the parameters in the system of ODEs are not the same as the parameters in the multiscale model.

#### 2.5.2. Problematic Issues in Strategies Relying on Canned Methods

The previous approaches for parameter fitting of the multiscale model with age are all relying on canned methods. The two main strategies are the ones worked out in [48,50]. In [48], the long-term approximation is used for the solution of the multiscale model equations and Levenberg–Marquardt is used as a canned method. One drawback of such an approach is that it is limited to the multiscale model under treatment. In addition, the analytical approximation would change when various multiscale models are introduced and the elaborative derivations would need to be carried for each one, with restrictions that are incorporated by the approximation being used. Finally, as elaborated in [57], the use of a canned method is distancing the user away from having control over the main optimization procedure and the ability to tune it from the programming standpoint.

## 3. Results

Having described the newer and significantly more efficient methods for parameter estimation relative to [57], we present the new results obtained for both the biphasic model [26] and multiscale models [47–50]. We first provide a basic illustration with the mutliscale model in which run-time and performance comparisons between methods are generated. Then, in Appendix B, for each type of model, some examples are described. The results are presented using a newer (efficient) version of the user-friendly simulator that we have initially developed in [55,57] for both biphasic and multiscale models. We start from the biphasic model in Appendix B and end with the multiscale model in Appendix C. The simulator with a GUI is freely available at http://www.cs.bgu.ac.il/~dbarash/Churkin/SCE/Efficient/Parameter_Estimation (the efficient version, with the option to either select the biphasic or the multiscale model).

For a basic comparison between all relevant parameter estimation methods, we apply our new methods on the difficult case of the retrieved data points that was also used for this purpose in [57] to compare our efficient methods with the previous ones. Results were obtained after a few minutes instead of the several hours that was reported in [57], making our tool practical also for difficult cases. As in [57], we fitted the four treatment parameters *κ*, *ε*_*s*_, *ε*_*α*_ and *γ* and all other parameters were selected with the values of Table 2.

We show in Table 3 the different values of those four parameters and sum of squared-errors fitted with the various methods (new efficient ones vs. previously published ones) to the data emanating from a patient. In the rightmost column, we fitted the long-term approximation with the retrieved data points using the scipy.optimize.curve_fit method, which is a Python implementation of a simple Levenberg–Marquardt scheme as a canned method. The next column to the right are the values obtained previously by the use of Levenberg–Marquardt along with the numerical method to solve the model equations as outlined in [57]. In the left columns are the values obtained by our new efficient methods. The small differences assure us that the significant efficiency achieved, thereby making our simulator a practical and useful tool, did not result in less accuracy.

**Figure 2.**
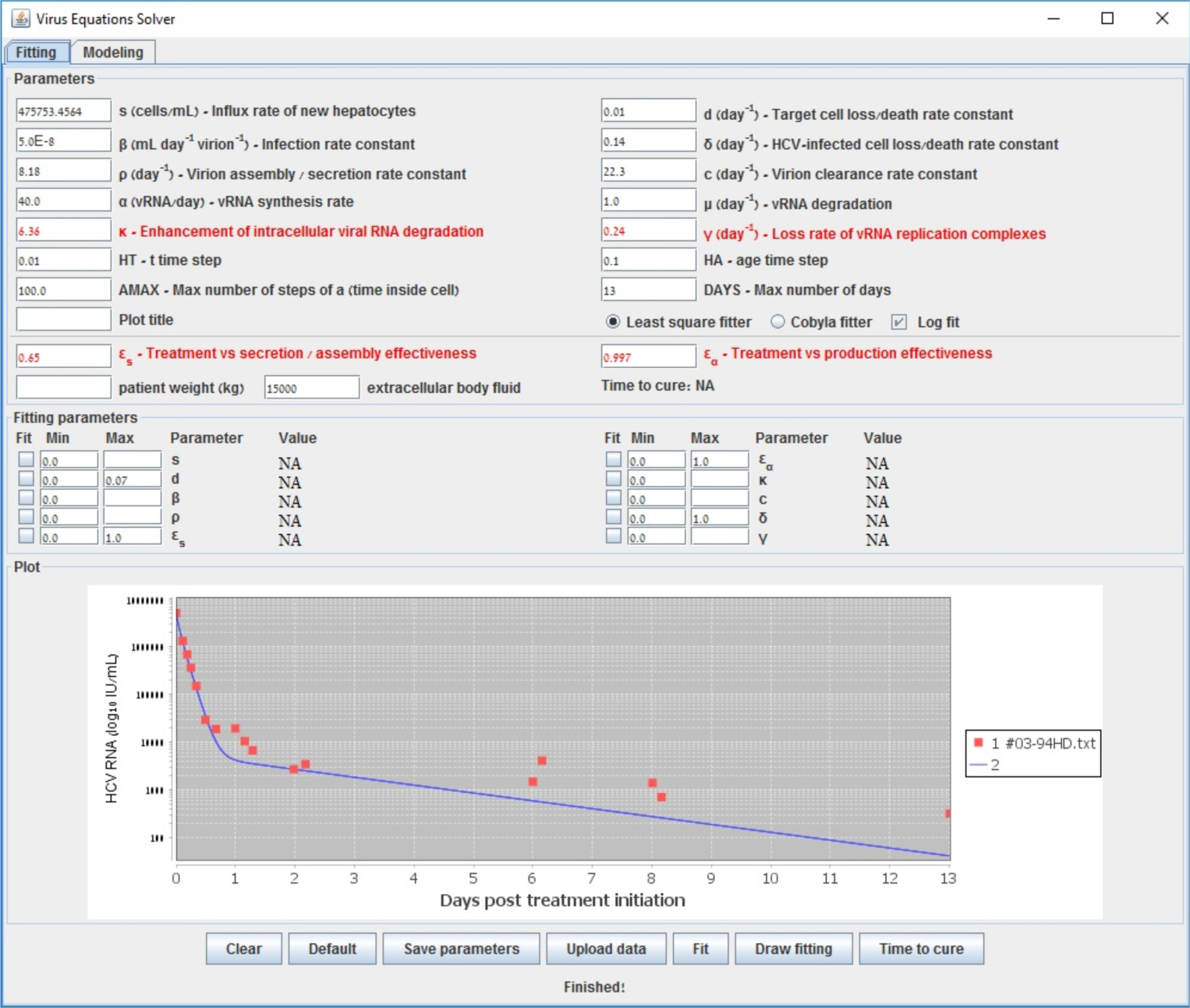
Start fit that emanates from data of a patient reported in [48]. The fitting curve corresponds to default parameters before fitting with our methods. The multiscale model is used.

**Figure 3.**
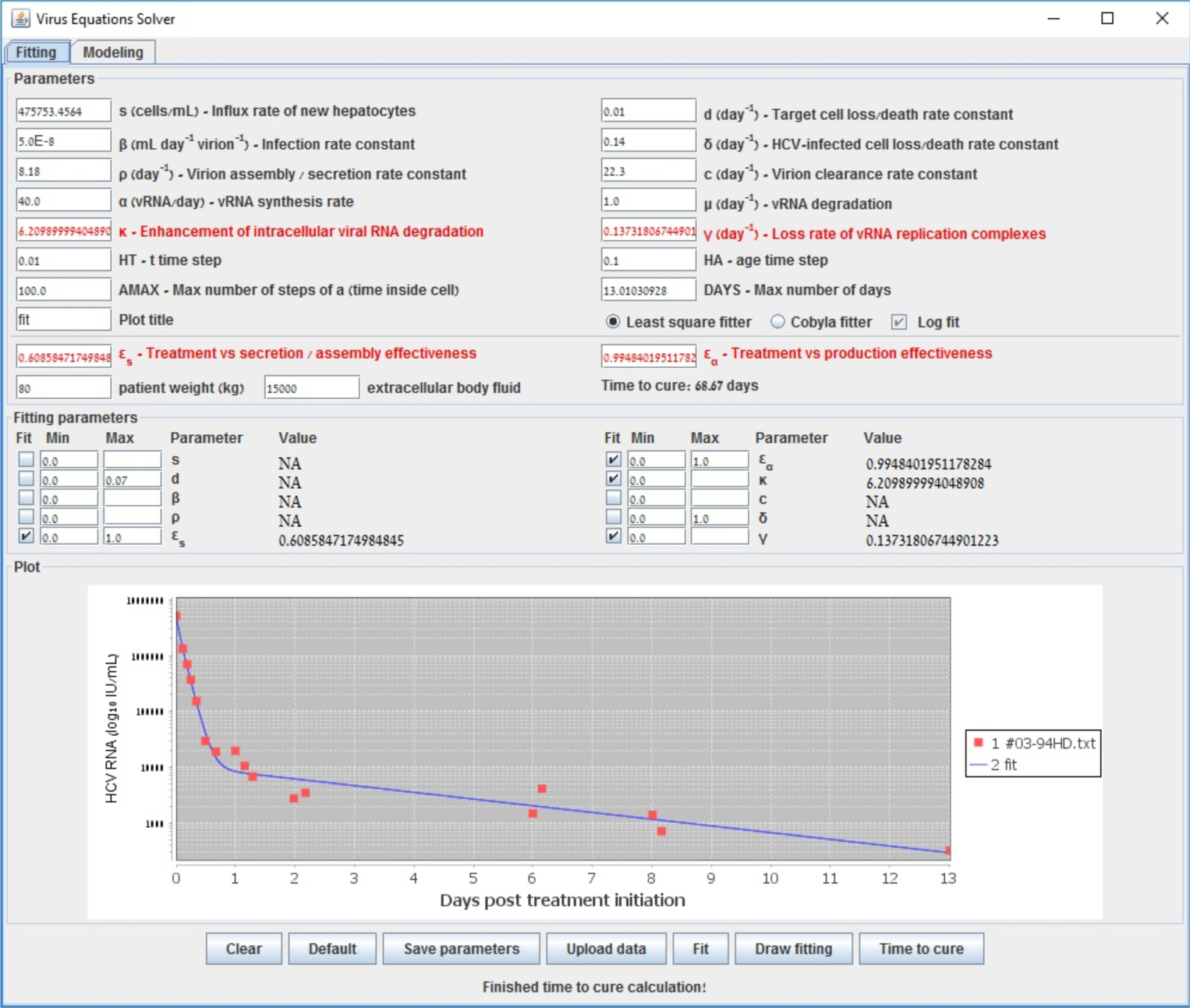
End fit using Gauss–Newton (LSF) that emanates from data of a patient reported in [48].

**Figure 4.**
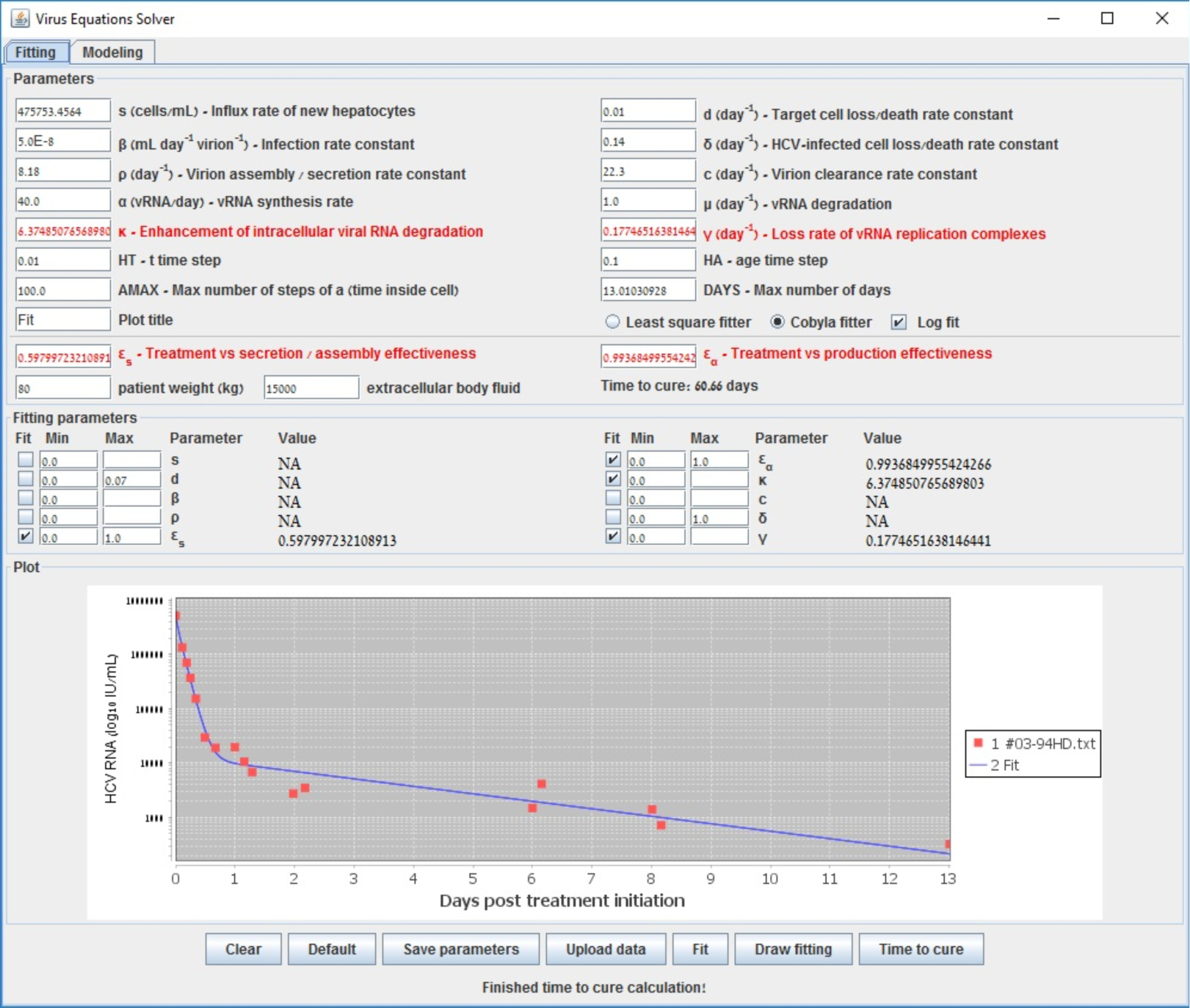
End fit using COBLYA that emanates from data of a patient reported in [48].

**Figure 5.**
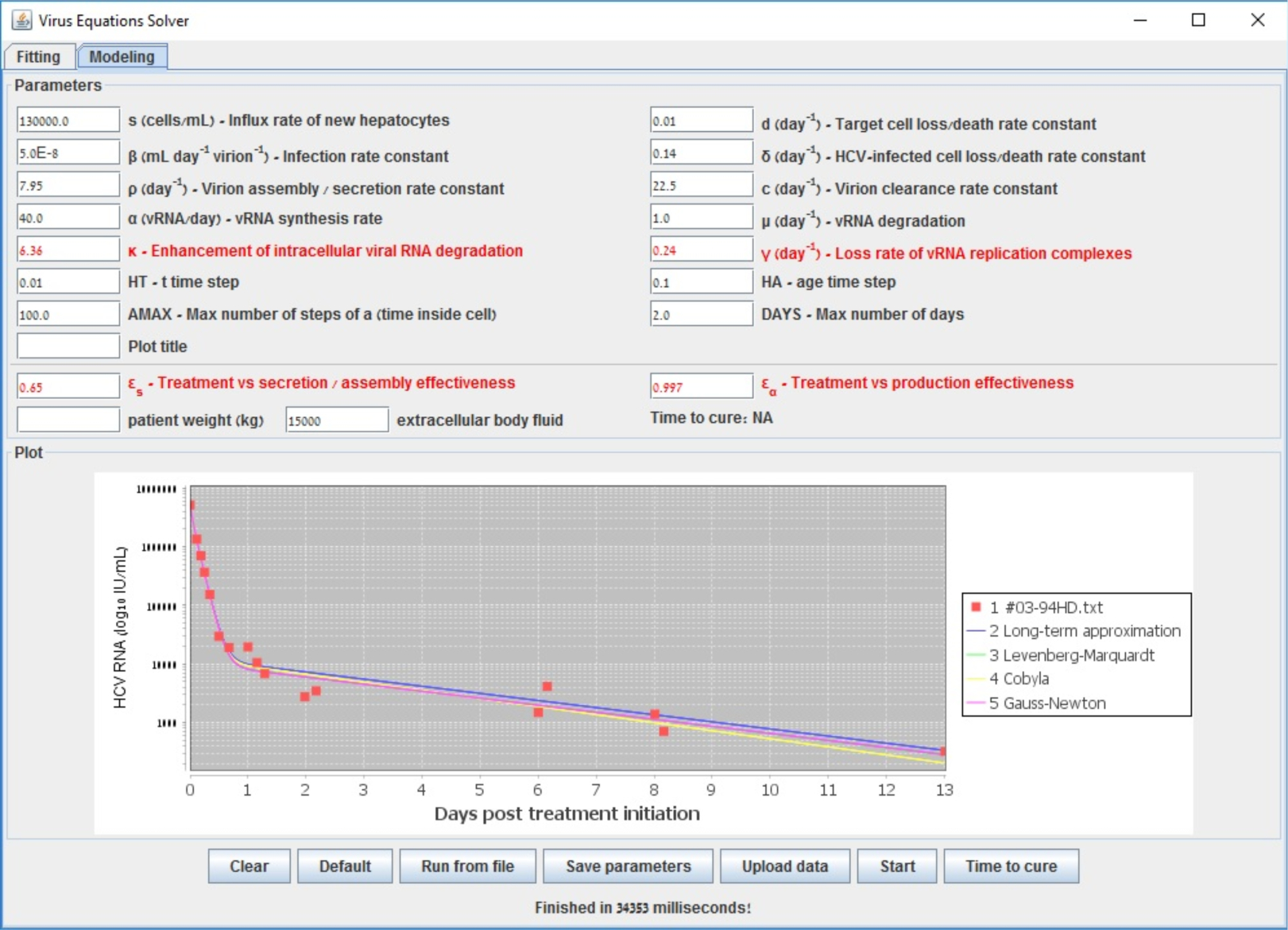
Comparison between the line fits of different methods inside the simulator window for the retrieved data points of patient HD that was reported in [48].

**Figure 6.**
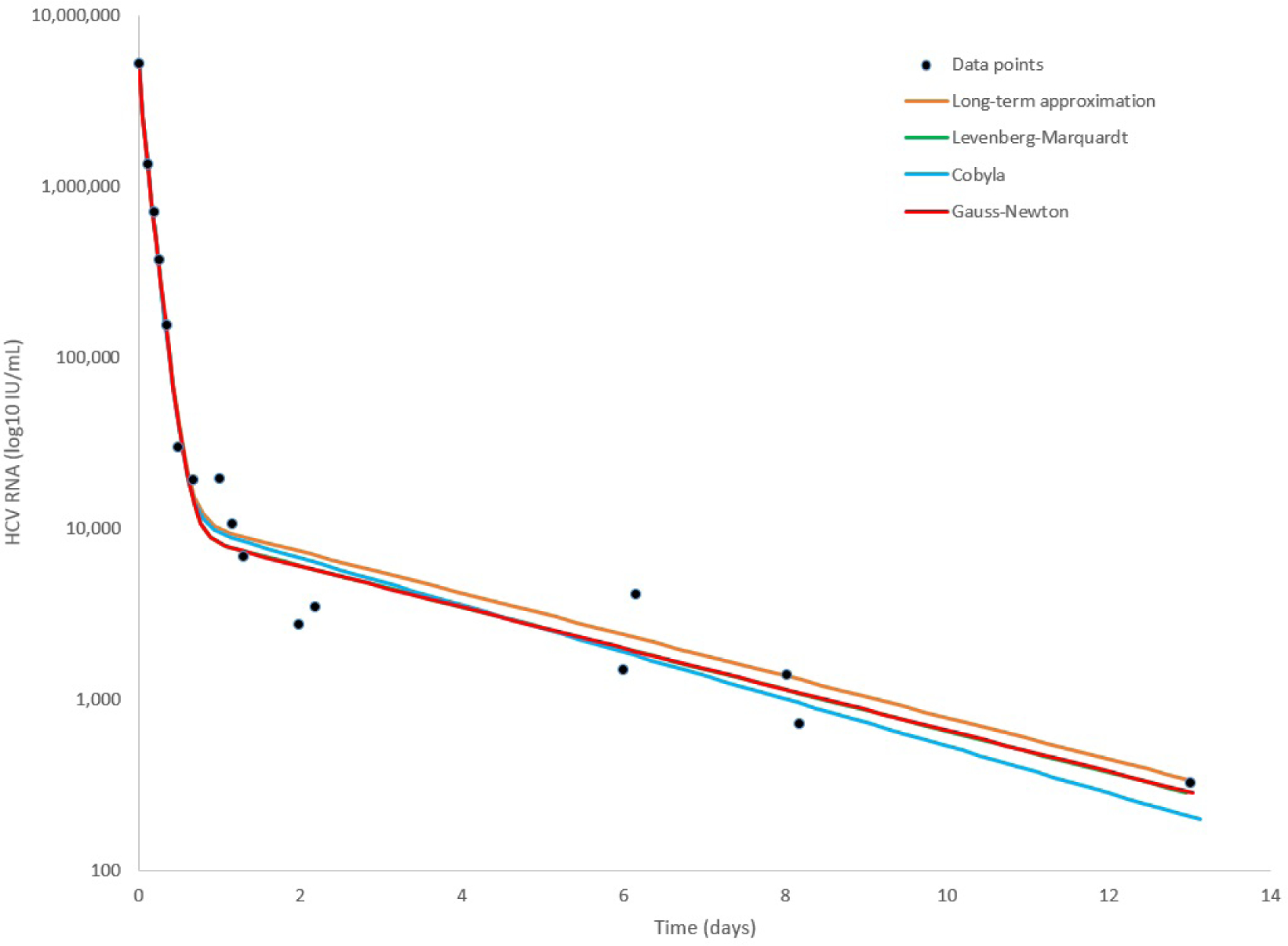
Comparison between the line fits of different methods for the retrieved data points of patient HD that was reported in [48].

To further illustrate the tool we provide, we show in Figure 2 the starting configuration after the data was inserted as input. The shown fitting curve is the one for default parameters (not considering data points) before running any fitting method. In Figures 3 and 4, the final results are shown when selecting LSF and COBYLA, respectively. In Figures 5 and 6, we present the curves of all methods shown in the same simulator window and in a separate graph, to which Table 3 corresponds.

## 4. Discussion

A practical and user-guided automatic procedure for parameter estimation is an important goal to achieve for mathematical models that are based on differential equations. It enables users to test a variety of fitting scenarios, either for the model calibration or model calibration with validation, by inserting different available data points of patients used for the fitting and fixed parameter values. The motivation is to use the parameters obtained by the fitting procedure to perform successful predictions for other data, where other data are data of new patients that form initial conditions to the model and successful predictions mean that the solution of the model equations yields a correct extraction of important quantities such as time to cure. In the context of viral dynamic models, even a simple model such as the biphasic model [26] that is beneficial to be tested by users requires a nonlinear method for the least squares minimization because a linear method is not sufficient [57]. The development of more complicated models such as viral dynamic models that consider intracellular viral RNA replication, namely age-structured PDE multiscale models to study viral hepatitis dynamics during antiviral therapy [47–50], presents a need for even more sophisticated strategies that perform parameter estimation while solving the model equations simultaneously. Efficient methods as developed herein are crucial such that the parameter estimation can be performed in a reasonable time.

From the parameter estimation standpoint, as previously outlined [57] and briefly mentioned in the Introduction, multiscale models are even more challenging than the biphasic model. Not only is conducting a search in at least a 10-parameter search space more difficult than in a 4-parameter search space, but also the task of solving the model equations themselves and how to connect the equations solution to the optimization procedure requires more sophistication. Previously, this was approached in [48] by using the long-term approximation along with a canned method for Levenberg–Marquardt, and in [50] by the method of lines and then employing Matlab’s 4th order Runge–Kutta solver along with a canned method available in Matlab called *fmincon* for the optimization. While these strategies work sufficiently well for specific cases because of their use of canned methods, they are problematic from the standpoint of the user’s capability to access and control them. Thus far, to the best of our knowledge, no specific source code for viral hepatitis kinetics besides our initial attempt at [57] (more general software such as DUNE, DuMuX, and UG4 for the solution of PDE models are available at [66–68] and would be worthwhile exploring in the future) has been released free of charge for the benefit of the community and while these strategies were described coherently in the context of presenting multiscale models, they were not intended to provide to the user a comprehensive solution of their own. There is clearly a need to provide the user with a free of charge simulator that is effortless to operate and a code that can be accessed for dissemination and future development. Furthermore, it should be practical in running time and allow inserting constraints for the parameters that need be estimated, which is not available in our initial attempt of [57] because of reverting to the standard non-constrained Levenberg–Marquardt method for the optimization and encountering numerical precision problems that were difficult to detect when developing the complete strategy for parameter estimation in our initial attempt.

The strategy we presented herein is a direct continuation to [57] and requires no canned methods utilization. It works directly on the multiscale model equations, preparing them in advance for the optimization procedure by taking their derivatives with respect to the parameters, in contrast to solving them first by an analytical approximation or performing the method of lines as a first step. For the solution of the model equations, the Rosenbrock method described in [55] is employed, as was shown to be advantageous in comparison to other solution schemes in [56]. For the constrained optimization procedure, as a departure from [57], either the Gauss–Newton or COBYLA are employed in full (not as a canned method) such that the user has access to the source code at each point in the procedure. Both Gauss–Newton and COBYLA are significantly more efficient in their constrained optimization procedure relative to the Levenberg–Marquardt employed in [57]. More complicated patient cases that took several hours of run time simulation in [57] (19.48 h reported in Table 3 for Levenberg–Marquardt) are now calculated in a few minutes (3.23 min reported in Table 3 for Gauss–Newton) on a standard PC, and simpler cases that took several minutes are now performed in seconds. Thus, the obtained results are much faster to compute than the existing solutions without sacrificing accuracy. The whole method is provided in a form of a model simulator with a user-friendly GUI, letting the user insert parameter constraints.

We note by passing that the aforementioned general software DUNE, DuMuX, and UG4 [66–68] are written in C++ using the MPI library allowing for massively parallel evaluations in the context of HPC, which might allow significantly more extended data sets to consider in the future. Thus, High Performance Computing (HPC) might also be an option for future development.

The code is open source and is divided into several packages: two fitter packages and a third default package with solver (Solver.java) class and GUI (GUI.java) class. The default package also contains different helper classes, like a class with all parameters and adapter classes to define the objective function for the fitters. The code is flexible and it is easy to add any new model solver class or any new parameters fitting class, library, or package. We use adapter design pattern to connect between the model solver and the parameters fitting algorithm. Thus, to add a new solver or fitter, one should add the solver/fitter code to the project and implement the adapter class that matches the interface of the model solver to the objective function interface of the parameters fitting method. In addition, one should change 2–3 rows in the GUI.java class to make use of a new solver/fitter from the GUI interface.

## 5. Conclusions and Future Work

The efficient methods described herein make the simulator a practical tool that is distributed free of charge for the benefit of the community and the dissemination of viral hepatitis models. Furthermore, the methods for parameter estimation employed can conceptually be used in other mathematical models in biomedicine.

Future work would include the development of the code in several directions. First, the code can be made more modular such that the modeler can easily implement the method for a different model or a modified version of one of these models. In this way, portability of the method to other models can be achieved such that a significant modification of the code is not needed as a consequence of a change in the model, ensuring that the modification is relatively straightforward. Second, at present, individual fits to individual time course profiles is available, which is useful when one wants to describe viral dynamics within one patient. The code can be developed for use also for fitting the in vitro time course profiles of pooled patient datasets. Thus, as future work, having the option to import and fit the models to repeated/multiple measurements would be useful.

From the numerical perspective, it might be possible to try weak methods for the solution of the model equations such as finite elements, finite volumes, or discontinuous Galerkin as described in [63,64]. The two time scales might present different challenges as compared to PDEs that are dependent on time and space in their partial derivatives. Independently, much of the computations are present in the optimization stage as compared to the solution stage and therefore efforts centered on the model equations solution could focus on simplified strategies, if at all possible, for the benefit of gaining more efficiency.

Finally, machine learning methods can be used to improve parameter estimation. There are already enough patient cases, as more than 250 patients have been modeled, which can be used to prepare the data for the parameter estimation of our simulator. Machine learning can then be used for outliers’ removal, replacement of the incorrect and missed data with the correct one (currently done manually), and correction of the data for the parameters and time to cure estimation. The machine learning algorithm can then be integrated with the parameter estimation method to yield an overall improved procedure.

## Figures and Tables

**Table 1.** The 12 parameters of the model.

*s* (cells mL^−1^)	Influx rate of new hepatocytes
*d* (d^−1^)	Target cell loss/death rate constant
*β* (mL d^−1^ virion^−1^)	Infection rate constant
*δ* (d^−1^)	HCV-infected cell loss/death rate constant
*ρ* (d^−1^)	Virion assembly/secretion rate constant
*c* (d^−1^)	Virion clearance rate constant
*α* (vRNAd^−1^)	vRNA synthesis rate
*μ* (d^−1^)	vRNA degradation
*κ*	Enhancement of intracellular viral RNA degradation
*γ* (d^−1^)	Loss rate of vRNA replication complexes
*ε*_*s*_	Treatment vs. secretion/assembly effectiveness
*ε*_*α*_	Treatment vs. production effectiveness

**Table 2.** Default parameters that are used herein. Parameter *s* comes from Equation (9), taking $\bar{V}$ as the max Virions value.

*α*	40 d^−1^	*β*	5 × 10^−8^ mL d^−1^
*c*	22.3 d^−1^	*δ*	0.14 d^−1^
*μ*	1 d^−1^	*d*	0.01 d^−1^
*ρ*	8.18 d^−1^	*s*	$(\bar{\nabla }\beta c+dc)/(\beta N)\text{ cells }/\text{mL}$

**Table 3.** Values of the parameters when fitted to the patient digitized data. The rightmost column has the values when the retrieved data points are fitted to the long-term approximation as in [57]. The left columns contain the fitted parameter values by our efficient methods. Except for the rightmost column, all methods are combined with the Rosenbrock numerical scheme. The fixed parameters have the values shown in Table 2. Run-time comparison is reported in seconds in the last row.

	Gauss-Newton (LSF)	COBYLA	Levenberg-Marquardt	Long-Term
*ε*_*s*_	0.609	0.598	0.602	0.600
*ε*_*α*_	0.995	0.994	0.995	0.994
*κ*	6.210	6.375	6.219	6.160
*γ* (d^−1^)	0.137	0.177	0.139	0.140
accuracy (sum error^2^)	0.538	0.582	0.538	0.587
run-time (s)	194	3698	70118	<1

**Figure A1.**
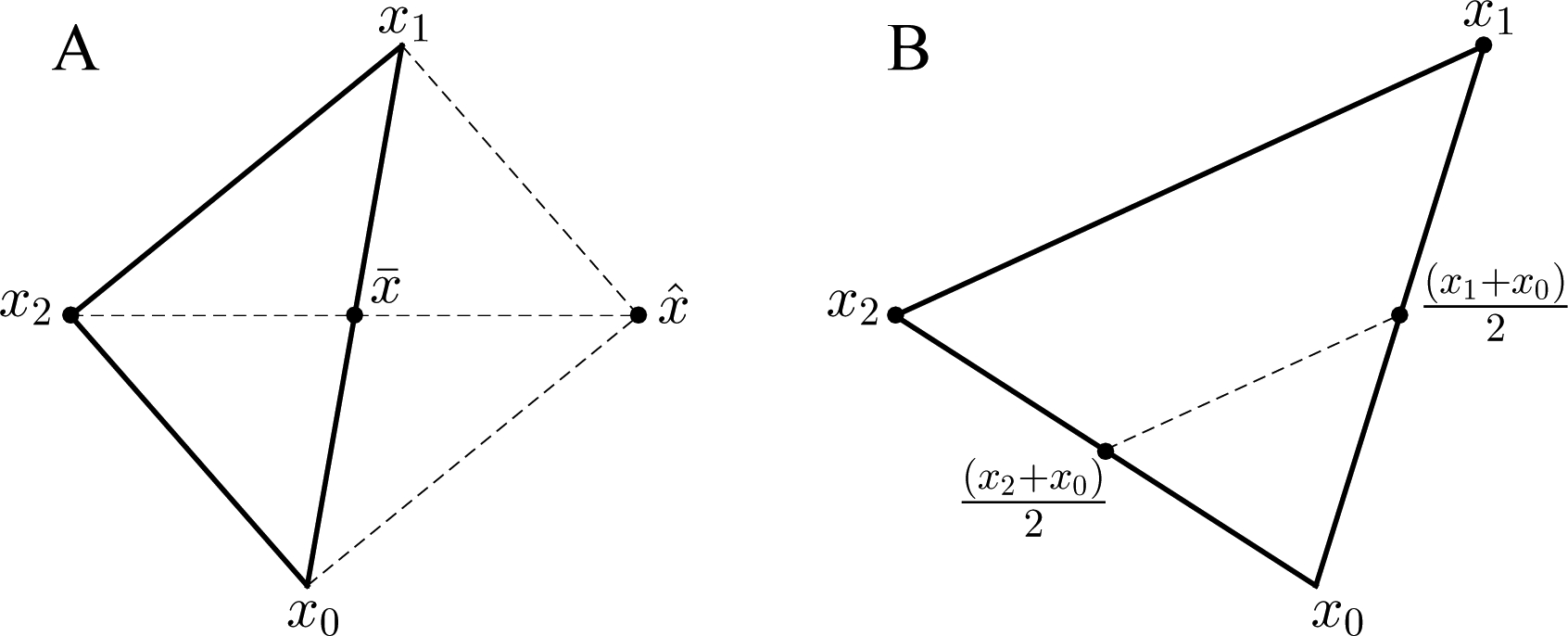
Illustration of the original simplex method. The points *x*_0_, *x*_1_, and *x*_2_ form the initial simplex. (**A**) The point $\bar{x}$ is the midpoint of the line joining *x*_0_ and *x*_1_, and $\hat{x}$ is the reflection of *x*_2_ through this line. If $f(\hat{x})<f\left(x_{1}\right),x_{2}$ is replaced by $\hat{x}$, shifting the location of the simplex. (**B**) If $f(\hat{x})\geq f\left(x_{1}\right),x_{2}$ is replaced with (1/2)(*x*_2_ + *x*_0_) and *x*_1_ is replaced with (1/2)(*x*_1_ + *x*_0_), reducing the volume of the simplex.

**Figure A2.**
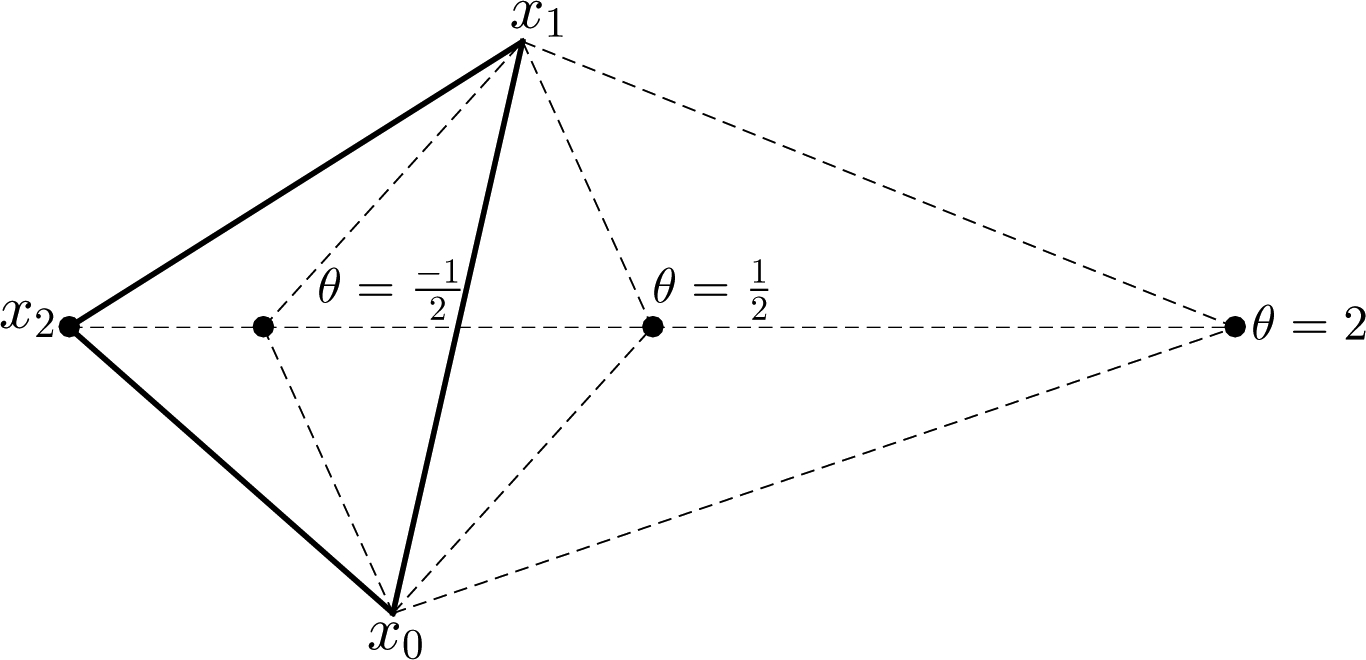
Illustration of the Nelder–Mead method. The points *x*_0_, *x*_1_ and *x*_2_ form the initial simplex. The vertex *x*_2_ is replaced with a vertex of the form $x_{\text{new }}=\bar{x}+\theta \left(\bar{x}-x_{2}\right)$. If $f(\hat{x})<f\left(x_{0}\right),\theta =2$, if $f\left(x_{0}\right)\leq f(\hat{x})<f\left(x_{n-1}\right),\theta =1/2$, and if $f\left(x_{n-1}\right)\leq f(\hat{x}),\theta =-1/2$.

**Figure A3.**
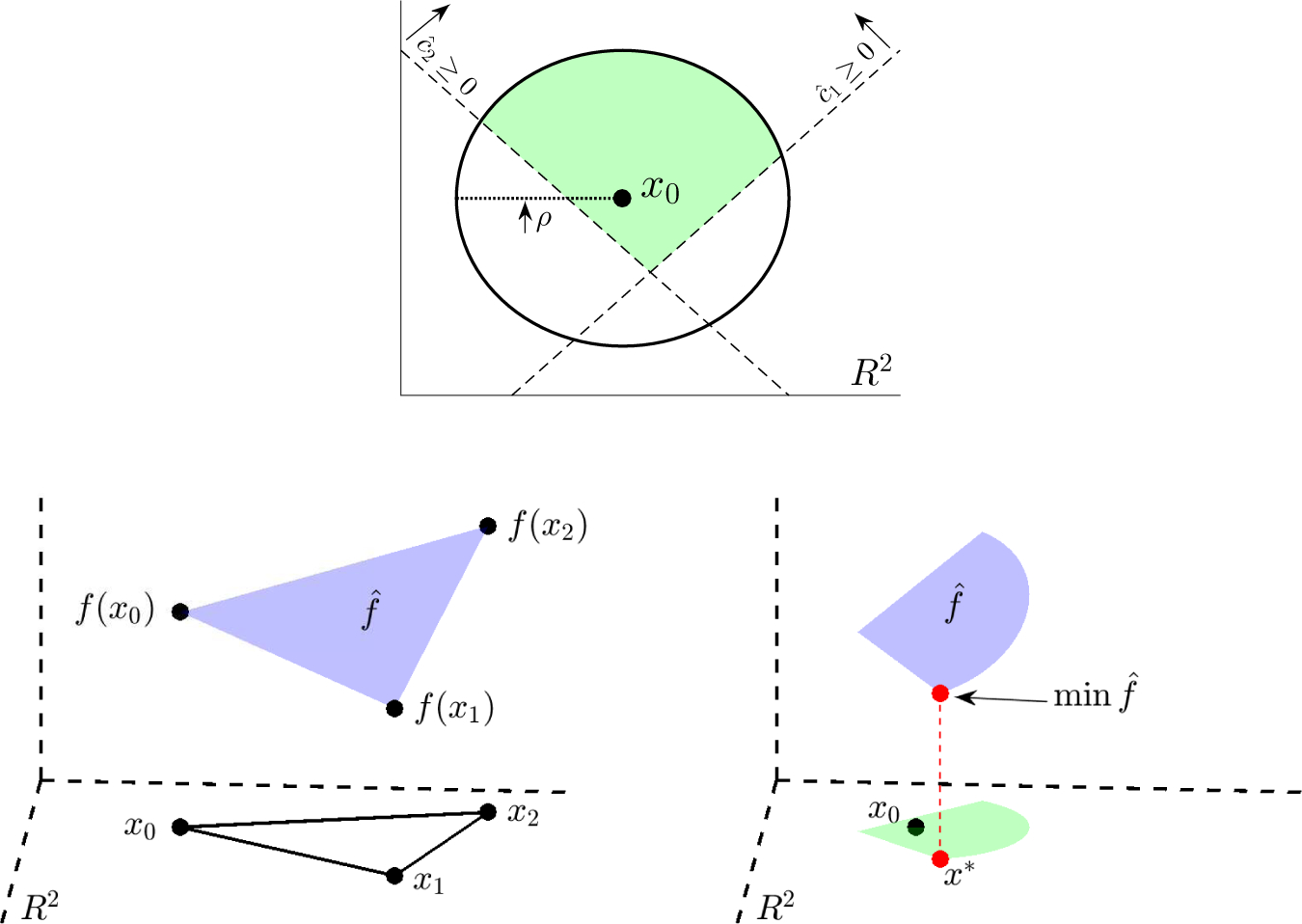
Minimization of $\hat{f}$ The candidate vertex x* is computed by minimizing $\hat{f}(x)$ subject to the constraints *ĉ*_1_, *ĉ*_1_ ≥ 0 within the trust region ∥*x* − *x*_0_∥_2_ ≤ *ρ*. (top) The region of optimization (green) is the intersection of the trust region ∥*x* − *x*_0_∥_2_ ≤ *ρ* with the half planes defined by the affine constraints *ĉ*_1_ ≥ 0 and *ĉ*_2_ ≥ 0. (bottom left) The function, $\hat{f}$, to be minimized is represented graphically by the plane (blue) passing through points (*x*_0_, *f*(*x*_0_)), (*x*_1_, *f*(*x*_1_)), (*x*_2_, *f*(*x*_2_)). (bottom right) The vertex x* is defined to be the point within the region of optimization (green) at which $\hat{f}$ (blue) is minimized.

**Figure A4.**
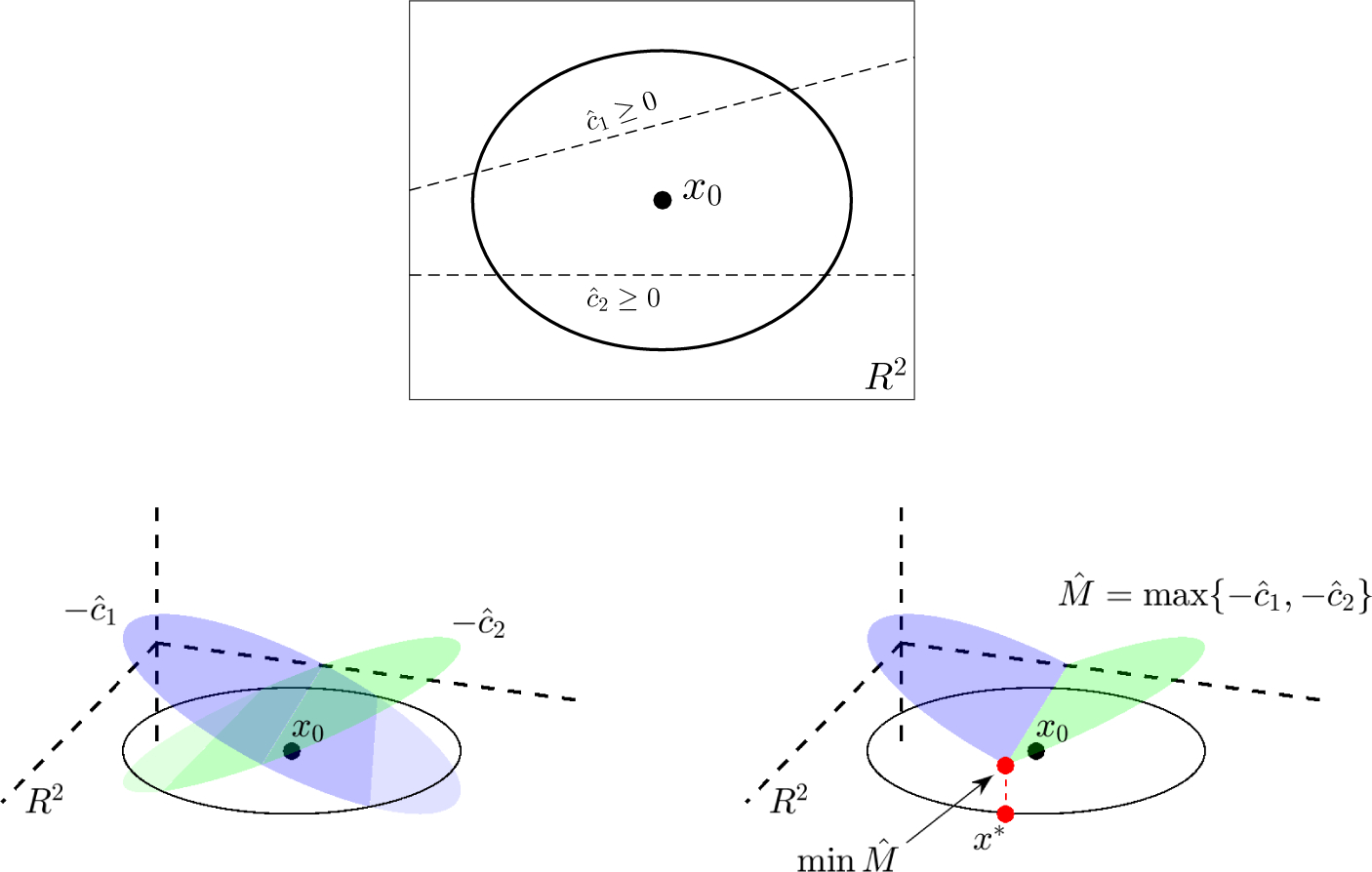
Minimization of $\hat{M}$ Should the constraints *ĉ*_*i*_(*x*) ≥ 0 be inconsistent with one another within the trust region ∥*x* − *x*_0_∥_2_ ≤ _*ρ*_, the candidate vertex x* is chosen to minimize $\hat{M}≔\text{max}\left\{-{\hat{c}}_{i}(x):i=1,\ldots ,m\right\}$. (top) The constraints *ĉ*_1_(*x*) ≥ 0 and *ĉ*_2_(*x*) ≥ 0 are inconsistent within the region ∥*x* − *x*_0_∥_2_ ≤ *ρ*. (bottom left) Graphs of the affine functions −*ĉ*_1_(*x*) (blue) and −*ĉ*_2_(*x*) (green). (bottom right) The vertex x* is defined to be the point within the trust region (black circle) at which $\hat{M}$ is minimized.

**Figure A5.**
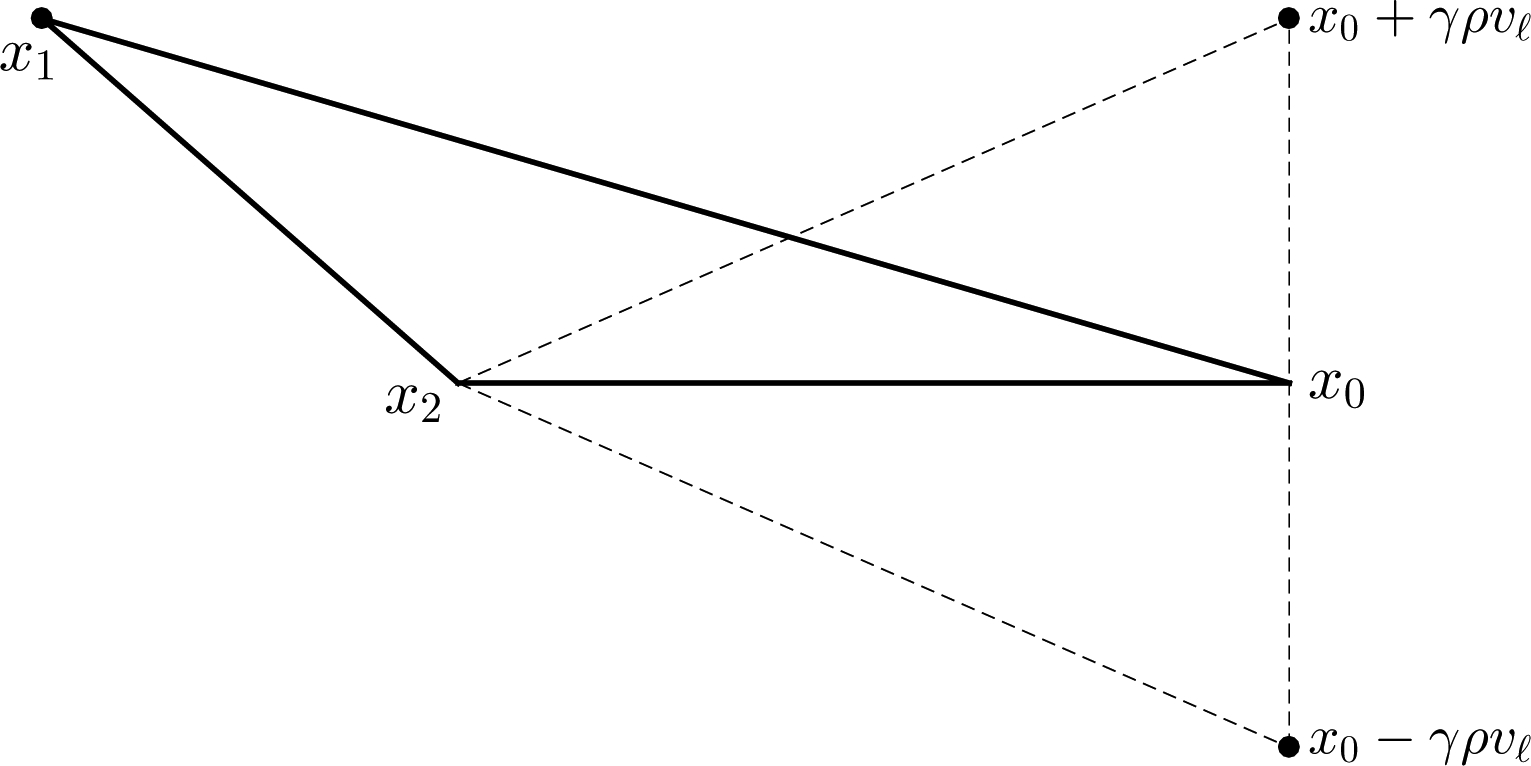
Illustration of the new vector *x*^Δ^, generated to improve the shape of the simplex. The vertex *x*_1_ is replaced with either $x^{\Delta }=x_{0}+\gamma \rho v_{l}$ or $x^{\Delta }=x_{0}-\gamma \rho v_{l}$, whichever point results in a smaller value of $\hat{\Phi }$.

**Figure A6.**
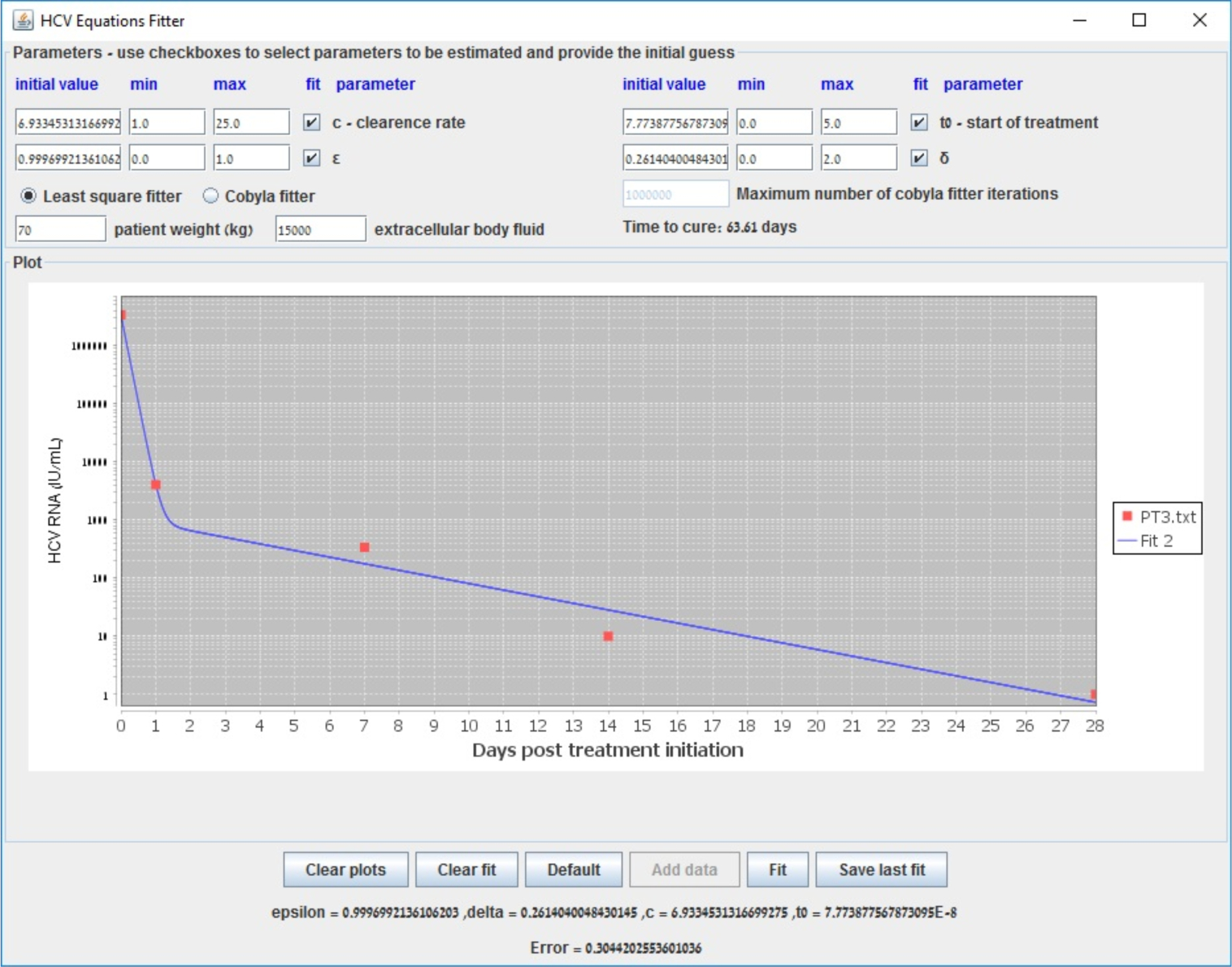
Biphasic model fitting example with data taken from [36] of a patient who was treated with mavyret. The LSF method (default) is recommended for use.

**Figure A7.**
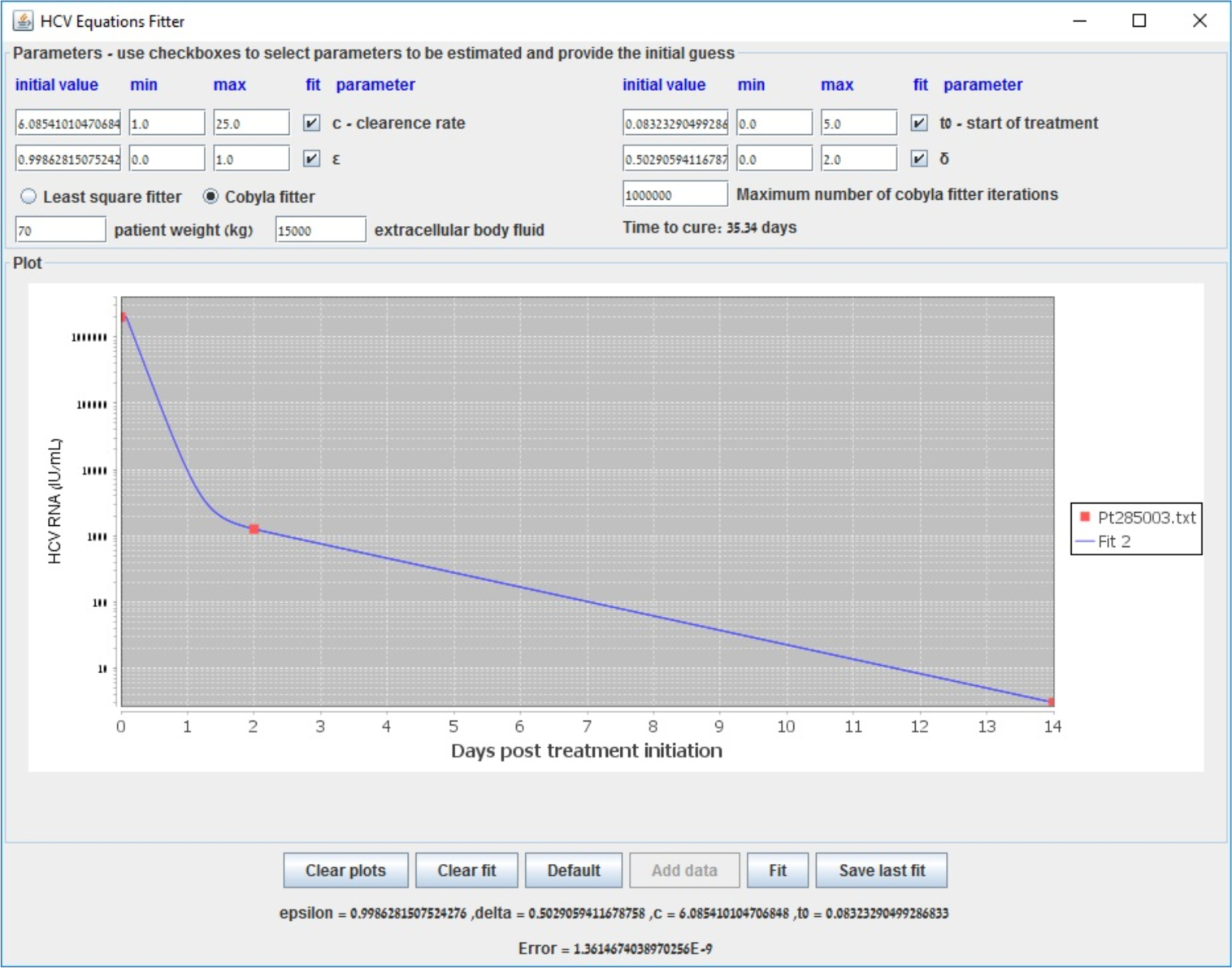
Biphasic model fitting example with data taken from [36] of a patient who was treated with epclusa. In this particular case, COBYLA was selected instead of LSF and succeeded to yield a fit.

**Figure A8.**
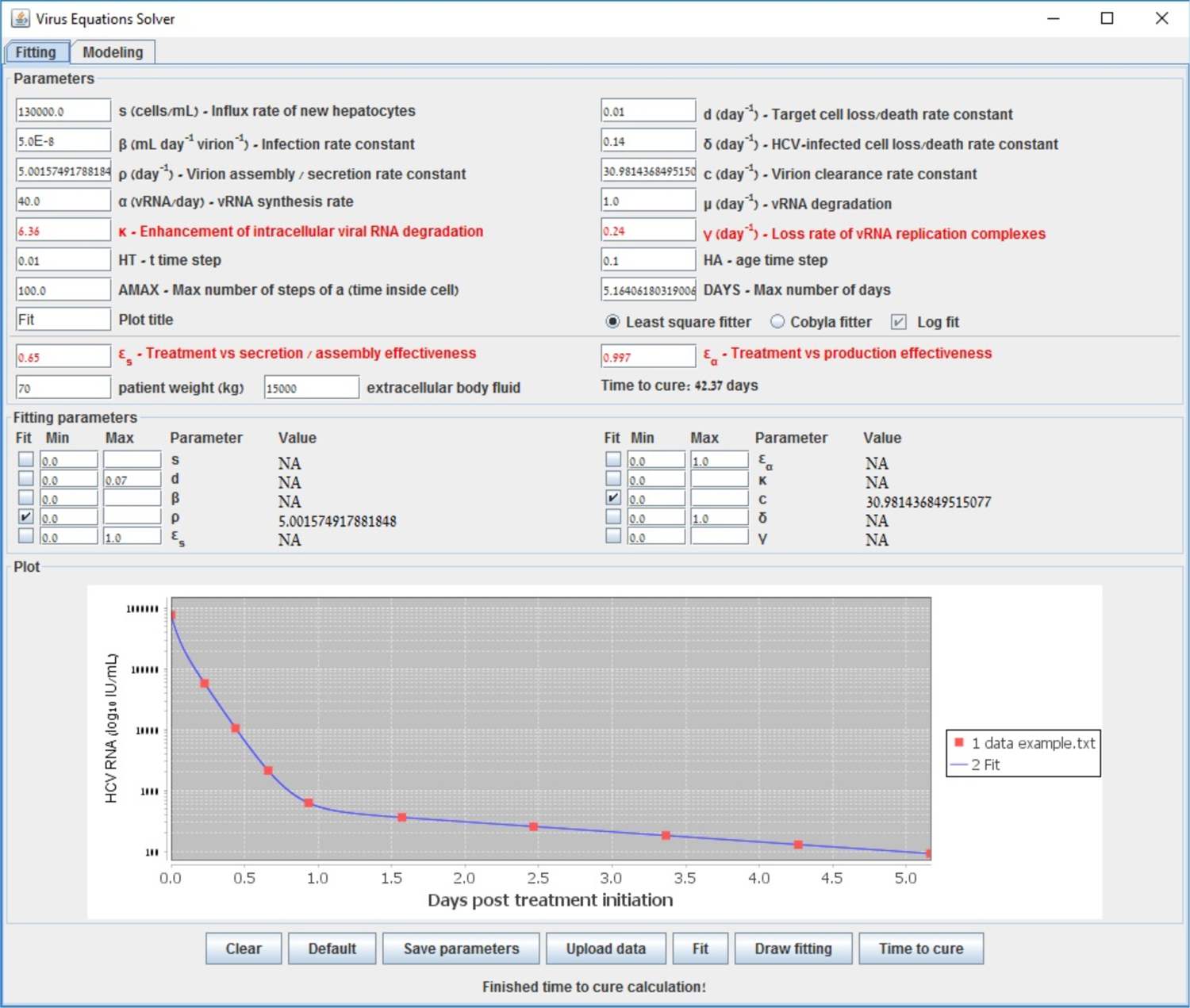
Fitting the parameters *c* and *ρ* of the multiscale model to generated data points using the LSF method.

**Figure A9.**
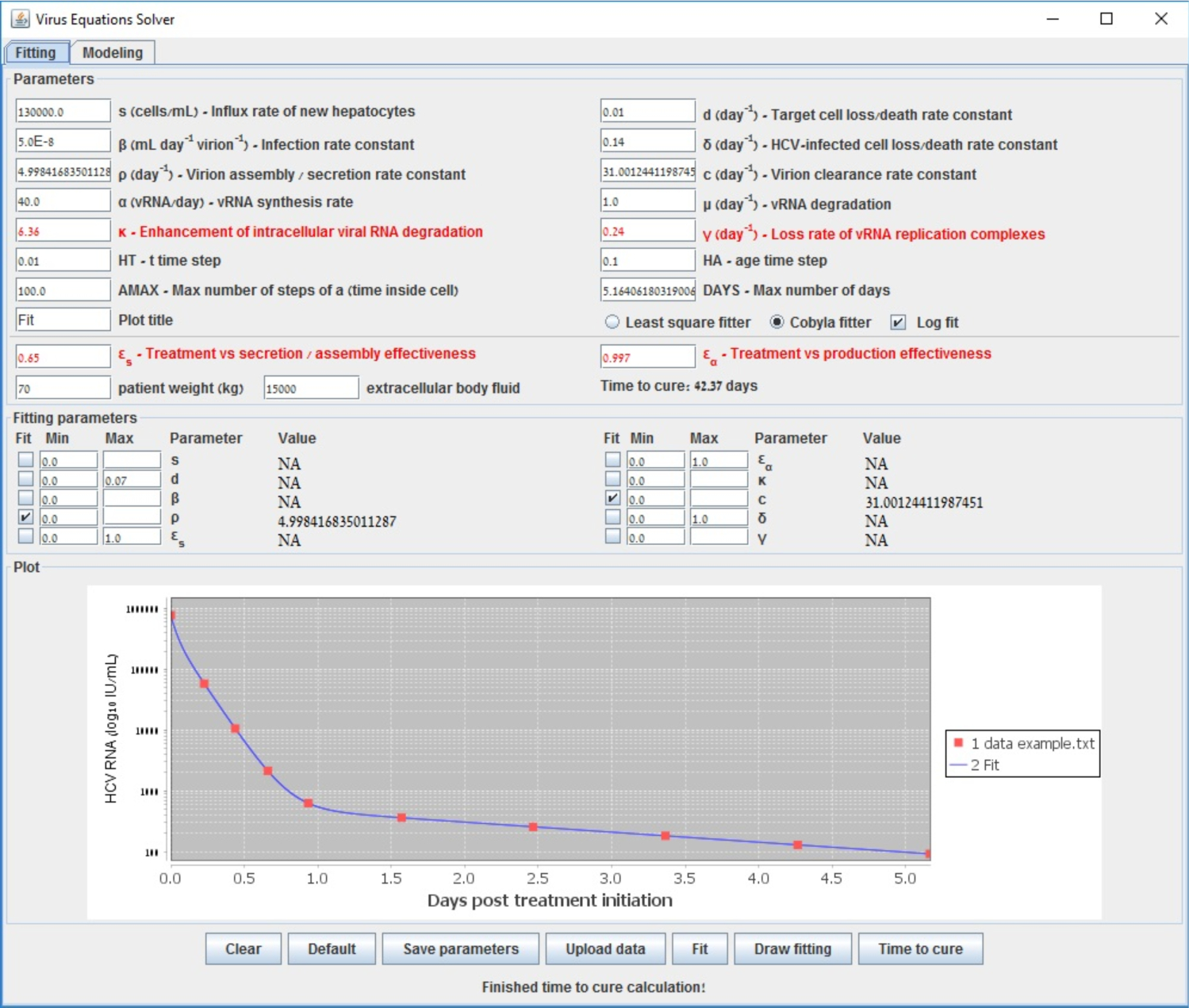
Fitting the parameters *c* and *ρ* of the multiscale model to generated data points using the COBYLA method.

## Appendix A. Details of the COBYLA Method

The original simplex method, devised by Spendley, Hext, and Himsworth [61,69], seeks to advance a simplex towards a minimizer of $f(x):{\mathbb{R}}^{n}\to \mathbb{R}$ by reflecting vertices at which *f* is large across opposite faces of the simplex in the hopes of reducing *f*. If the initial simplex has vertices *x*_0_, *x*_1_, …, *x*_*n*_, ordered so that *f*(*x*_0_) ≤ *f*(*x*_1_) ≤ … ≤ *f*(*x*_*n*_), then *x*_*n*_ is the vertex at which *f* is largest, and is considered for revision. The vector $\hat{x}$, defined below, is proposed as a replacement:

(A1) $$\hat{x}≔\frac{2}{n}\left(\overset{n-1}{\underset{i=0}{\sum }}x_{i}\right)-x_{n},$$

(A2) $$=\frac{1}{n}\left(\overset{n-1}{\underset{i=0}{\sum }}x_{i}\right)+\left(\frac{1}{n}\left(\overset{n-1}{\underset{i=0}{\sum }}x_{i}\right)-x_{n}\right),$$

(A3) $$≔\bar{x}+\left(\bar{x}-x_{n}\right).$$

Note that the vector $\bar{x}$ is the centroid of the convex hull of the points *x*_0_, *x*_1_, …, *x*_*n*−1_, so that $\hat{x}$ is the reflection of the vertex through the face of the simplex opposite *x*_*n*_. As such, the volume of the simplex is preserved if the vertex exchange $x_{n}\to \hat{x}$ is made, with the swap occurring if $f(\hat{x})<f\left(x_{n-1}\right)$. If $f(\hat{x})$ is comparable to *f*(*x*_*n*_), the assumption is made that *f* is minimized in the area between *x*_*n*_ and $\hat{x}$, so that the change $x_{n}\to \hat{x}$ simply bypasses this region. To access this interior region, the simplex is rescaled without replacing any of the vertices with $\hat{x}$. The optimal vertex, *x*_0_, is left alone, and each vertex *x*_*k*_, for *k* = 1, …, *n*, is replaced with (1/2)(*x*_0_ + *x*_*k*_). Both such changes to the simplex are illustrated in the case *n* = 2 in Figure A1.

The Nelder–Mead method [70] expands on this basic simplex method, removing much of the inefficiency that arises when rescalings result in a small simplex that takes longer to converge to the function’s minimizing region. It does so by moving the vertex *x*_*n*_ to a new point along the line joining *x*_*n*_ and $\hat{x}$, strategically chosen to reduce *f* as much as possible. A generic expression for the new vertex, *x*_new_, is now,

(A4) $$x_{\text{new }}≔\bar{x}+\theta (\bar{x}-x_{n}),$$

where *θ* > 0 may be different at each iteration. In the case of a linear *f*, we have that

(A5) $$f\left(x_{\text{new }}\right)=f(\bar{x})+\theta \left(f(\bar{x})-f\left(x_{n}\right)\right)\leq f(\bar{x}),$$

where the last equality above follows from the fact that *f*(*x*_*n*_) ≥ *f*(*x*_*k*_) for all *k* = 0, 1, …, *n* − 1, which implies $f(\bar{x})\leq f\left(x_{n}\right)$. Regarding the linear case as a proxy for the more general case, we expect such a vector *x*_new_ to decrease the value of *f*, if *θ* is chosen well. One particular implementation bases the choice of *θ*—and thus the new vertex—on the value of $f(\hat{x})$ relative to the value of *f* at other vertices. The new vertex, $\overset{ˇ}{x}$, is defined as such:

$$\left\{\begin{array}{lll} 2\hat{x}-\bar{x} & \text{ if }f(\hat{x})<f\left(x_{0}\right), & \text{(A}6\text{a)}\\ \frac{1}{2}(\bar{x}+\hat{x}) & \text{ if }f\left(x_{0}\right)\leq f(\hat{x})<f\left(x_{n-1}\right), & \text{(A}6\text{b)}\\ \frac{1}{2}\left(x_{n}+\bar{x}\right) & \text{ if }f\left(x_{n-1}\right)\leq f(\hat{x}). & \text{(A}6\text{c)} \end{array}\right.$$

The choices in Equations (A6a)–(A6c) are obtained by taking *θ* = 2, (1/2), (−1/2), respectively, in Equation (A4), and are depicted in Figure A2. If $f(\hat{x})<f\left(x_{0}\right)$, as in Equation (A6a), then *f* decreases so significantly along the line from *x*_*n*_ to $\hat{x}$ that choosing a new vertex, $2\hat{x}-\bar{x}$, even further down the line is presumed to result in a greater reduction. If $f\left(x_{0}\right)\leq f(\hat{x})<f\left(x_{n-1}\right)$, as in Equation (A6b), the reduction in *f* is less significant, and the new vertex is placed between $\hat{x}$ and the face of the simplex opposite *x*_*n*_. If $f\left(x_{n-1}\right)\leq f(\hat{x})$, as in Equation (A6c), the reduction in *f* at $\hat{x}$ is minimal, and $\overset{ˇ}{x}$ is placed between *x*_*n*_ and the face of the opposite simplex, as placement of the vertex near $\hat{x}$ is not warranted.

The COBYLA algorithm is used to minimize an objective function $f(x):{\mathbb{R}}^{n}\to \mathbb{R}$ subject to the set of m∈ℕ constraints,

(A7) $$\left\{c_{i}(x)\geq 0:i=1,2,\ldots ,m\right\},$$

where $c_{i}(x):{\mathbb{R}}^{n}\to \mathbb{R}$ for each *i* = 1, ⋯, *m*. As derivatives are omitted from the algorithm entirely, no smoothness assumptions are required for the functions *f*, *c*_*i*_; they must simply be well-defined on ${\mathbb{R}}^{n}$. After generating the initial simplex from an initial guess as to the location of the minimizer, the optimal vertex is identified and labeled *x*_0_. In this case, a vertex of the simplex is considered optimal if Φ(*x*_0_) ≤ Φ(*x*_*k*_), for *k* = 1, ⋯, *n*, where the *x*_*k*_ are the other *n* vertices of the simplex, and Φ(*x*) is defined by

(A8) $$\Phi (x)=f(x)+\mu {\left[\text{max}\left\{-c_{i}(x):i=1,\cdots ,m\right\}\right]}_{+},$$

with [*x*]_+_ = max{*x*, 0}, and *μ* ≥ 0 a constant parameter. The optimality of a point *x* is thus affected by both the value of *f*(*x*) and how closely it satisfies the constraints in Equation (A7). If *c*_*i*_(*x*) ≥ 0 for all *i* = 1, ⋯, *m*, then Φ(*x*) = *f*(*x*), but if at least one constraint is violated, then Φ(*x*) > *f*(*x*), lessening the “worth” of the point *x* as an approximation to the minimizer. From there, each iteration of the algorithm generates a new candidate vertex, designed to either replace an existing vertex with one that decreases Φ(*x*) or improves the shape of the simplex. The shape of the simplex is particularly crucial in this algorithm because its vertices are used to define linear programming problems, from which new candidate vertices designed to improve the optimality condition are derived. Specifically, if {*x*_*k*_ : *k* = 0, ⋯, *n*} are the vertices of the current simplex, we let $\hat{f}(x):{\mathbb{R}}^{n}\to \mathbb{R}$ be the unique affine function that passes through points $\left(x_{k},f\left(x_{k}\right)\right)\in {\mathbb{R}}^{n+1}$, and, analogously, we let ${\hat{c}}_{i}(x):{\mathbb{R}}^{n}\to \mathbb{R}$ be the unique affine function that passes through the points (*x*_*k*_, *c*_*i*_(*x*_*k*_)). Should the shape of the simplex be “acceptable”—in a way to be defined later—a new candidate vertex is chosen to improve optimality by minimizing $\hat{f}(x)$ subject to the constraints {*ĉ*_*i*_(*x*) ≥ 0}. Should the simplex be of an unacceptable shape, the linear programming problem may be ill-defined or fail to provide a reasonable approximation to the functions *f*(*x*), *c*_*i*_(*x*). If this newly generated vector improves the value of Φ, it replaces a vertex of the simplex. This process continues until a pre-determined final trust region radius, which represents the desired accuracy of the approximation to the minimizer, is achieved.

The algorithm takes as inputs an initial guess, *x*_0_, as to the location of the minimizer, and the constants *ρ*_beg_, *ρ*_end_ > 0, which represent the initial and final trust region radii. Additionally, *μ* is set to zero. At the start, the initial simplex is generated from *x*_0_ and *ρ*_beg_. The vector *x*_0_ is one vertex, with the other vertices, *x*_*k*_, *k* = 1, …, *n*, defined by *x*_*k*_ = *x*_0_ + *ρ*_beg_*e*_*k*_, for *k* = 1, …, *n*. Here, *e*_*k*_ is the *k*-th coordinate vector. After each *x*_*k*_ is generated, *f*(*x*_*k*_) is computed, and the labels of the vectors *x*_0_ and *x*_*k*_ are swapped if *f*(*x*_*k*_) < *f*(*x*_0_), to ensure that *f* is minimized at *x*_0_. After the initial simplex is defined, the algorithm proceeds to advance the simplex at each iteration by either generating a new candidate vertex, denoted *x**, to decrease Φ(*x*), or an alternate vertex, denoted *x*^Δ^, to improve the shape of the simplex. Each iteration begins by ensuring that *x*_0_ is the optimal vertex–and relabeling vertices if it is not–before assessing the suitability of the simplex. For this, denote by *σ*^*k*^ the Euclidean distance from the vertex *x*_*k*_ to the face of the simplex opposite *x*_*k*_, and, by *η*^*k*^, the length of the segment joining *x*_*k*_ to *x*_0_. The simplex is deemed to have an acceptable shape if and only if *σ*^*k*^ ≥ *αρ* and *η*^*k*^ ≤ *βρ* for all *k* = 1, …, *n*, where *α*, *β* satisfy 0 < *α* < 1 < *β*. These conditions prevent the development of flat, degenerate simplices, which would result in poorly-formulated linear programming problems. If the simplex is of an unacceptable shape, a vertex *x*^Δ^ is generated; otherwise, a candidate vertex *x** is computed.

The iteration immediately following formation of the initial simplex always generates a vertex *x**, as the initial simplex satisfies *σ*^*k*^ = *η*^*k*^ = *ρ*_beg_ for all *k* = 1, …, *n*, and is thus always acceptable. The loop that generates such an *x** in general is described below; in the very first iteration, *ρ* = *ρ*_beg_. The vector *x** is generated by minimizing $\hat{f}(x)$ subject to the constraints in Equation (A7) and the trust region condition,

(A9) $${\left\|x-x_{0}\right\|}_{2}\leq \rho ,$$

as illustrated in Figure A3. Should the constraints in Equations (A7) and (A9) be inconsistent with one another, the candidate *x** is chosen by minimizing the “greatest constraint violation” function, $\hat{M}(x)$, defined by,

(A10) $$\hat{M}(x)≔\text{max}\left\{-{\hat{c}}_{i}(x):i=1,\ldots ,m\right\}$$

subject to the trust region constraint in Equation (A9). This process is illustrated in Figure A4. If there exist multiple *x** that satisfy $\hat{M}\left(x^{*}\right)=\text{min}\left\{\hat{M}(x):{\left\|x-x^{\left(0\right)}\right\|}_{2}\leq \rho \right\}$, then the *x** that minimizes $\hat{f}(x)$ is chosen from among those that minimized $\hat{M}(x)$. If there are multiple such minimizers of $\hat{f}(x)$, then the one that minimizes ∥*x* − *x*_0_∥_2_ is chosen.

When the appropriate *x** is identified, the condition ${\left\|x^{*}-x_{0}\right\|}_{2}<\frac{1}{2}\rho$ is tested. If ${\left\|x^{*}-x_{0}\right\|}_{2}\geq \frac{1}{2}\rho$, the relative “size” of the parameter *μ* is evaluated. To this end, denote by $\bar{\mu }$ the smallest value of *μ* for which $\hat{\Phi }\left(x^{*}\right)\leq \hat{\Phi }\left(x_{0}\right)$, where

(A11) $$\hat{\Phi }(x)=\hat{f}(x)+\mu {\left[\text{max}\left\{-{\hat{c}}_{i}(x):i=1,\ldots ,m\right\}\right]}_{+}=\hat{f}(x)+\mu {\left[\hat{M}(x)\right]}_{+}.$$

If Equations (A7) and (A9) are consistent with one another, then *x** had been chosen to minimize $\hat{f}(x)$, and it follows that $\hat{\Phi }\left(x^{*}\right)=\hat{f}\left(x^{*}\right)\leq \hat{f}\left(x_{0}\right)=\hat{\Phi }\left(x_{0}\right)$. In this case, $\bar{u}=0$. Should Equations (A7) and (A9) be inconsistent, then *x** had been chosen to minimize $\hat{M}(x)$ as in Equation (A10), implying that $\hat{M}\left(x_{0}\right)-\hat{M}\left(x^{*}\right)\geq 0$. If $\hat{M}\left(x_{0}\right)-\hat{M}\left(x^{*}\right)=0$, then $\hat{M}$ has at least two minimizers, and *x** had been chosen to minimize $\hat{f}(x)$, implying $\hat{\Phi }\left(x^{*}\right)\leq \hat{\Phi }\left(x_{0}\right)$ from Equation (A11). If $\hat{M}\left(x_{0}\right)-\hat{M}\left(x^{*}\right)>0$, there exists a $\bar{\mu }$ sufficiently large to guarantee $\hat{f}\left(x_{0}\right)-\hat{f}\left(x^{*}\right)+\mu \left(\hat{M}\left(x_{0}\right)-\hat{M}\left(x^{*}\right)\right)>0$, even if $\hat{f}\left(x_{0}\right)-\hat{f}\left(x^{*}\right)<0$, implying $\hat{\Phi }\left(x^{*}\right)\leq \hat{\Phi }\left(x_{0}\right)$. for $\mu \geq \bar{\mu }$ If the current value of *μ* satisfies $\mu \geq \frac{2}{3}\bar{\mu }$, then *μ* is considered “sufficiently large” and its value is left alone. Otherwise, *μ* is increased to 2*μ*.

If *μ* is increased, it may no longer be the case that *x*_0_ is optimal, in the sense that there may exist some *k* between 1 and *n* for which Φ(*x*_*k*_) < Φ(*x*_0_). From the form of Φ in Equation (A8), this reversal of the original order relation Φ(*x*_*k*_) ≥ Φ(*x*_0_) as *μ* increases can only occur if [*M*(*x*_0_)]_+_ > [*M*(*x*_*k*_)]_+_, with *M*(*x*) defined as:

(A12) $$M(x)≔\text{max}\left\{-c_{i}(x):i=1,\ldots ,m\right\}.$$

If *x*_0_ is no longer optimal, the process returns to the start of the loop, from which it first rearranges the labeling of the vertices so as to label the optimal one *x*_0_. No change was made to the vertices of the simplex beyond this relabeling, and if the simplex had been acceptable previously, it will remain acceptable. In this case, another candidate vertex *x** will be computed with respect to the new *x*_0_. The process of generating *x**, increasing *μ*, and then relabeling the vertices can only happen a finite number of times; the labels of the vertices *x*_0_ and *x*_*k*_ are only switched if *M*(*x*_0_) > *M*(*x*_*k*_), meaning that the value of *M* decreases each time a vertex exchange is made. Thus, increasing *μ* can only change the order relation between the values Φ(*x*_*k*_) until the optimal vertex *x*_0_ also satisfies *M*(*x*_0_) = min{*M*(*x*_*k*_) : *k* = 0, …, *n*}, at the latest.

If *μ* is sufficiently large and *x*_0_ is optimal, or once these conditions have been achieved, the possible replacement of one vertex with the new candidate vertex *x** is considered. The values of *f*(*x**) and *c*_*i*_(*x**) are computed, and the simplex is revised to incorporate *x** as a vertex if Φ(*x**) < Φ(*x*_0_), or if such a revision will improve acceptability of the simplex. The choice of which existing vertex should be replaced with *x** is determined by the predicted effect of the change on the acceptability conditions and the volume of the simplex. For this, consider the quantity ${\bar{\sigma }}^{k}$, defined to be the Euclidean distance from *x** to the face of the simplex opposite *x*_*k*_. If *x*_*k*_ is replaced with *x**, but all other vertices are left unchanged, the volume, *V**, of the new simplex is related to the volume, *V*^*k*^, of the original simplex by the formula,

(A13) $$V^{*}=\frac{{\bar{\sigma }}^{k}}{\sigma^{k}}V^{k}.$$

This follows from the standard formula for the volume of a simplex in ${\mathbb{R}}^{n}$, which yields $V^{k}=(1/n)V^{H}\sigma^{k},V^{*}=(1/n)V^{H}{\bar{\sigma }}^{k}$, where *V*^*H*^ is the volume of the convex hull of the *n* points *x*_0_, *x*_1_, …, *x*_*j*−1_, *x*_*j*+1_, …, *x*_*n*_. It is considered advantageous for a change in the vertices to increase the volume of the simplex, and, as such, vertices *x*_*k*_ for which ${\bar{\sigma }}^{k}>\sigma^{k}$ are sought for replacement by *x**. Specifically, consider the set *J* defined by

(A14) $$J≔\left\{j:{\bar{\sigma }}^{j}\geq \sigma^{j}\right\}\cup \left\{j:{\bar{\sigma }}^{j}\geq \alpha \rho \right\},$$

thus consisting of indices *j* for which *x*^*j*^ could be replaced with *x** either without decreasing the volume of the simplex or without disturbing the acceptability condition *σ*^*j*^ ≥ *αρ*. To consider the effect of a change on the acceptability condition *η*^*k*^ ≤ *βρ*, note that, if a vertex is replaced by *x**, the optimal vertex of the new simplex will be either the previous optimal vertex, *x*_0_, or the vertex *x** itself. Denoting the new optimal vertex by ${\bar{x}}_{0}$, if *J* is nonempty, let l∈ℕ be defined as

(A15) $$l≔\text{min}\left\{k\in \mathbb{N}\cap [1,n]:{\left\|x_{k}-{\bar{x}}_{0}\right\|}_{2}=\text{max}\left\{{\left\|x_{j}-{\bar{x}}_{0}\right\|}_{2}:j\in J\right\}\right\},$$

so that $x_{\left(l\right)}$ is the vertex with smallest index at a maximal distance from the optimal vertex. If ${\left\|x^{l}-{\bar{x}}_{0}\right\|}_{2}>\delta \rho$, for 1 < *δ* ≤ *β*, then *x*_*k*_ is replaced by *x**. If these conditions are not met, the simplex is revised regardless, as long as either Φ(*x**) < Φ(*x*_0_) or there exists an index *k* for which ${\bar{\sigma }}^{k}>\sigma^{k}$—that is, if Φ is smaller at the new candidate vertex, or a change increases the simplex’s volume. In this case, *x*_0_ is replaced by $x_{l}$, where l is now defined as

(A16) $$l≔\text{min}\left\{k\in \mathbb{N}\cap [1,n]:{\bar{\sigma }}^{k}/\sigma^{k}=\text{max}\left\{{\bar{\sigma }}^{j}/\sigma^{j}:j\in \mathbb{N}\cap [1,n]\right\}\right\}.$$

Note that, even though the simplex is updated whenever Φ(*x**) < Φ(*x*_0_), the vertex to discard is chosen based on the effect the change will have on the volume and acceptability of the simplex, with no regard for the values of Φ at different vertices. This process almost always results in an update to the simplex, with an update failing to occur only if Φ is not decreased at *x**, no vertex swap would increase the volume of the simplex, and either *J* is empty or ${\left\|x_{j}-{\bar{x}}_{0}\right\|}_{2}\leq \delta \rho$ for all *j* = 1, …, *n*. The set *J* is empty if each hypothetical vector swap actually decreases the volume of the simplex and fails to maintain the first acceptability condition, ${\bar{\sigma }}^{j}\geq \alpha \rho$.

After potentially making a vector replacement, the progress made in reducing Φ as the simplex advances is examined. If sufficient progress is not made, the trust region radius, *ρ*, will be reduced. To determine if such a reduction should be made, the change in Φ at *x** is compared to the change in $\hat{\Phi }$. The condition

(A17) $$\frac{\Phi \left(x^{*}\right)-\Phi \left(x_{0}\right)}{\hat{\Phi }\left(x^{*}\right)-\hat{\Phi }\left(x_{0}\right)}\leq 0.1$$

is tested. If Cond. (A17) fails, then the improvement to Φ is at least 10% of the improvement to $\hat{\Phi }$. This improvement is considered significant enough, and the iteration returns to the start of the loop to generate a successive *x**, assuming the simplex is still acceptable. If Cond. (A17) fails, the improvement to *x** will be less than 10% of the improvement to $\hat{\Phi }$, and a decrease in the trust region radius is called for. Before the trust region radius is assessed, the acceptability of the simplex is checked. If the simplex is unacceptable, the iteration returns to the start of the loop, and generates an alternate vertex *x*^Δ^. If the simplex is acceptable, the condition *ρ* ≤ *ρ*_end_ is tested. If *ρ* ≤ *ρ*_end_, the final trust region radius has been reached, and the algorithm terminates. If *ρ* > *ρ*_end_, further advances to the simplex are required to reduce the trust region radius to its final value. The iteration returns to the start of the loop, and will generate a new candidate, *x**, as the acceptability of the simplex has already been verified. Before this, *ρ* and *μ* are updated. If *ρ* > 3*ρ*_end_, then *ρ* is decreased by half. If *ρ* ≤ 3*ρ*_end_, then *ρ* is set to *ρ*_end_ itself.

It is considered sensible to update *μ* whenever *ρ* is updated, as the value of *μ* can become quite large. A constraint function *c*_*i*_(*x*) is regarded as “significant” to Φ if *i* ∈ *I*, where

(A18) $$I≔\left\{i\in \mathbb{N}\cap [1,m]:c_{i}^{\text{min}}<(1/2)c_{i}^{\text{max}}\right\},$$

with $c_{i}^{\text{min}}$ and $c_{i}^{\text{max}}$ representing the minimum and maximum values of *c*_*i*_ at the vertices of the current simplex. If *I* is empty, *μ* = 0, and if *I* is nonempty, *μ* is set to the value,

(A19) $$\frac{\left\{{\text{max}}_{k=0,1,\ldots ,n}f\left(x_{k}\right)-{\text{min}}_{k=0,1,\ldots ,n}f\left(x_{k}\right)\right\}}{\text{min}\left\{{\left[c_{i}^{\text{max}}\right]}_{+}-c_{i}^{\text{min}}:i=1,\ldots ,m\right\}},$$

assuming that the quantity in Equation (A19) is less than the current value of *μ*.

If after generating the candidate vector *x** the condition ${\left\|x^{*}-x_{0}\right\|}_{2}<\frac{1}{2}\rho$ is met, much of the previously described process is omitted. The algorithm proceeds as it did after advancing the simplex and verifying Cond. (A17), by first checking the acceptability of the simplex and revising, if necessary, and then checking the condition *ρ* ≤ *ρ*_end_. As before, if *ρ* ≤ *ρ*_end_, the algorithm terminates, and, if *ρ* > *ρ*_end_, *ρ* and *μ* are updated as previously described and the process returns to the start of the loop to generate a new *x**.

It only remains to describe the process of updating the simplex to improve its acceptability. This is done by generating an alternate vertex, *x*^Δ^, to replace one of the vertices of the simplex. Recall that, if the simplex is unacceptable, then there either exists a j∈ℕ∩[1,n] such that *σ*^*k*^ < *αρ* or such that *η*^*k*^ > *βρ*. If the latter is true, define l∈ℕ∩[1,n] to be the index that satisfies

(A20) $$\eta^{l}=\text{max}\left\{\eta^{k}:k=1,\ldots ,n\right\}.$$

Otherwise, define l to be the index that satisfies

(A21) $$\sigma^{l}=\text{min}\left\{\sigma^{k}:k=1,\ldots ,n\right\}.$$

The vertex $x_{l}$ is then one that violates one of the acceptability conditions most egregiously, either by being the closest to the optimal vertex or the farthest from its opposite face. The vertex $x_{l}$ will be replaced by, *x*^Δ^, where *x*^Δ^ is defined by

(A22) $$x^{\Delta }=x_{0}\pm \gamma \rho v_{l},$$

where $v_{l}$ is the unit vector perpendicular to the face of the simplex opposite the outgoing vertex $x_{l}$, and *γ* ∈ (*α*, 1). The + or − is chosen to minimize $\hat{\Phi }$. The new vertex *x*_Δ_, illustrated in Figure A5, maintains the general position of *x*_*`*_ relative to the opposite face of the simplex, while satisfying *σ*^Δ^ = *η*^Δ^ = ∥*x*^Δ^ − *x*_0_∥_2_ = *γρ* ∈ (*αρ*, *βρ*). Even though the acceptability condition is not violated at the new vertex *x*^Δ^, acceptability may still be violated at other vertices. After replacing $x_{l}$ with *x*_Δ_, the process always generates a vertex of the type *x**, as opposed to conducting several replacements to improve acceptability. As described previously, the algorithm continues to advance the simplex by replacing current vertices with improvements of the form *x** and *x*^Δ^ until the condition *ρ* ≤ *ρ*_end_ is achieved.

## Appendix B. Parameter Estimation in the Biphasic Model

We begin with the standard model for HCV dynamics, the biphasic model of Neumann et al. [26]. Although the model is nonlinear, it can be solved analytically when assuming that the target cells *T* variable is constant. We incorporated the analytical solution described in [26] to our simulator and performed parameter estimation using a constrained optimization Gauss–Newton solver to solve the minimum least squares problem, which is more efficient and stable than the non-constrained nonlinear solver with a damping factor described in [57]. There can be instances in which the Gauss–Newton solver fails, although it is the simplest and most efficient, in which case the COBYLA solver should be used instead by selecting its option in the simulator or it can be used in all instances from the start. Figures A6 and A7 present fitting results from two patients (Pts). Figure A6 corresponds to Pt3 who was treated with mavyret [36] where LSF was selected for the optimization (default). In a case that corresponds to Pt285003 who was treated with epclusa [36] where LSF was selected for the optimization (default), a warning appeared because of a failure, after which Figure A7 corresponds to the same case, but this time COBYLA was selected for the optimization and succeeded to yield a fit. Our current method has recently been used in [71,72]. A webpage with user instructions is available at http://www.cs.bgu.ac.il/~dbarash/Churkin/SCE/Efficient/Parameter_Estimation/Biphasic.

## Appendix C. Parameter Estimation in the Multiscale Model

Extending to the multiscale model for HCV dynamics, we perform parameter estimation on the model taken from [48]. As previously described, in our simulator, we solved the model equations using the Rosenbrock method [55] and performed parameter estimation after preparing the derivative equations using a full implementation of our developed Gauss–Newton (LSF) and COBYLA methods that are suitable to our application domain without reverting to canned methods.

To illustrate the tool we provide, we first show in Figure A8 the result of fitting to generated data points from the default values of the parameters *c ρ*, when starting away from their real values (with initial guesses of *ρ* = 7.95, *c* = 22.5) and selecting the LSF method. The predicted values after running LSF (*ρ* = 5.0, *c* = 31.0) are very close to their real values (error of 2.58 · 10^−6^) and run-time was 40 s. In Figure A9, we show the result of the selecting the COBYLA method for the case as in the previous figure. The predicted values after running COBYLA are even slightly closer to their real values (error of 1.34 · 10^−8^) for this particular case and run-time was 109 s. Thus, starting from initial guesses that are far from the real values can be handled, indicating the robustness of our methods. Run-times of our methods were significantly faster than a run-time of 1680 s (with even a larger error of around 0.5 showing much less robustness) when using the Levenberg–Marquardt method that was implemented in [57]. A webpage with user instructions is available at http://www.cs.bgu.ac.il/~dbarash/Churkin/SCE/Efficient/Parameter_Estimation/Multiscale.
